# Running Constraints Shape Multifractal Movement Complexity Beyond Physiological and Mechanical Load: A Pilot Study

**DOI:** 10.3390/s26185881

**Published:** 2026-09-17

**Authors:** Andreia Teixeira, Catarina Abrantes, Daniel Santarém, Nuno Mateus, Jaime Sampaio

**Affiliations:** Department of Sports Science, Exercise and Health, Research Center in Sports Sciences, Health Sciences and Human Development (CIDESD), School of Life Sciences and Environment, University of Trás-os-Montes and Alto Douro, 5000-801 Vila Real, Portugal; andreiat@utad.pt (A.T.); abrantes@utad.pt (C.A.); danielrs@utad.pt (D.S.); nmateus@utad.pt (N.M.)

**Keywords:** multifractal analysis, nonlinear dynamics, temporal complexity, running, task constraints

## Abstract

**Highlights:**

**What are the main findings?**

- Running constraints produced distinct response profiles.
- HKR induced the highest cardiovascular demand.
- TR6% elicited the greatest mechanical load.
- Multifractal dynamics differed across running conditions.
- Temporal complexity was partly independent of load.

**What are the implications of the main findings?**

- Load metrics alone do not fully characterise running.
- Nonlinear metrics complement conventional load measures.
- Running constraints shape movement organisation.
- Complexity metrics may improve exercise monitoring.

**Abstract:**

Movement variability reflects adaptive neuromuscular reorganisation, but linear metrics alone cannot capture its temporal structure. This pilot study examined how running variations shape physiological and mechanical load responses using linear and nonlinear metrics. Fifteen recreationally active adults (31.4 ± 9.4 years; five females) performed six 90-s exercise conditions: high-knee running (HKR), tethered running (TER), shoulder-width running (SWR), core-board skipping (CBS), and treadmill running at 0% (TR0%) and 6% grade (TR6%). Heart rate (HR), muscle oxygenation (SmO_2_), and player load (PL) were recorded, and PL multifractal structure was quantified via wavelet leader analysis, validated against iterative amplitude-adjusted Fourier transform (IAAFT) and shuffled surrogates. HKR elicited the highest HR mean, while TR6% produced the greatest PL mean. Multifractal parameters—h(0), hmin, Dh (hmin), and left-tail width L—differed significantly across conditions (all *p* < 0.001), though overall spectral width (Δh) did not. Surrogate validation confirmed genuine temporal multifractality in 85/86 series. Load-adjusted regime analysis showed temporal complexity partly dissociable from load: HKR retained the highest scaling complexity beyond load, CBS combined lowest load with highest ordinal irregularity, and treadmill conditions were temporally constrained. HKR elicited the greatest physiological and mechanical demands, whereas CBS combined the lowest physiological load with the highest movement irregularity, demonstrating distinct exercise-specific response profiles. Given the modest, heterogeneous sample, these findings are presented as pilot evidence for this multi-metric approach, warranting confirmation in larger, sex-balanced cohorts.

## 1. Introduction

In running, movement variability reflects the adaptive capacity of the neuromuscular system to dynamically reorganise movement solutions across varying demands [1,2,3]. Even under controlled conditions, stride-to-stride fluctuations are present and functionally meaningful, rather than noise [4,5].

To capture these fluctuations, analytical approaches sensitive to both magnitude and temporal structure are required. Linear methods such as the mean, standard deviation, coefficient of variation, and root mean square quantify variability magnitude effectively but assume stationarity and linearity, treating deviations from the mean as random noise, rather than structured signal [6]. Nonlinear methods address these limitations by quantifying regularity, predictability, and hierarchical organisation of fluctuations. Sample entropy (SampEn) indexes signal regularity at a single temporal scale; higher values are conventionally interpreted as reflecting greater irregularity and, by extension, adaptive capacity, although this correspondence remains debated and should not be assumed a priori [7]. Multifractal analysis extends this by characterising scale-dependent variability simultaneously across multiple temporal scales, revealing how movement control operates from rapid neuromuscular adjustments to longer-term compensatory mechanisms [8]. Conceptually, a multifractal signature is generally taken to be consistent with cross-scale interactions, whereby fluctuations at coarser scales may constrain or modulate fluctuations at finer scales; this interpretation aligns with, but does not by itself confirm, multiplicative cascade and cross-scale interactivity accounts of biological movement organisation [9,10].

These approaches have been applied to heart rate variability [11], neural activity [12], neuromuscular control [13], and player displacement in team sports [14,15], as well as to coupled cardiac–neural dynamics under extreme physiological stress [16]. In running, nonlinear dynamics have been predominantly studied under treadmill conditions or in relation to fatigue, speed, and fitness level [6,17]. No study has simultaneously examined physiological and mechanical responses across ecologically distinct running variations using nonlinear approaches. This gap matters because mechanical and physiological systems are intrinsically coupled during exercise [5,18].

A further critical question is whether nonlinear movement characteristics remain distinguishable after accounting for conventional physiological and mechanical load magnitude, that is, whether running variations induce distinct temporal complexity regimes beyond differences in exercise intensity. This pilot study therefore aimed to examine acute physiological and mechanical responses across six running activities—high-knee running (HKR), tethered running (TER), shoulder-width running (SWR), core-board skipping (CBS), treadmill running at 0% grade (TR0%), and inclined treadmill running at 6% grade (TR6%)—using linear and nonlinear metrics. We hypothesised that (1) dynamic ground-based and constrained running conditions would produce distinct physiological and mechanical response profiles relative to treadmill-based running, with HKR and TER expected to elicit higher cardiovascular demand and TR6% expected to produce greater mechanical load; and (2) conditions imposing greater motor control demands (SWR, CBS) would produce wider multifractal spectral width (Δh) and greater left-tail asymmetry (L) relative to TR0%. We further expected these conditions to preserve or increase global scaling structure (DFA α, Higuchi fractal dimension) independently of load magnitude, so that greater local irregularity would not come at the expense of long-range temporal organisation.

This second hypothesis was grounded in degrees-of-freedom compensation accounts of motor control, whereby tasks that perturb habitual coordination solutions (e.g., an altered base of support and postural instability, as in SWR and CBS) are expected to increase local, fine-scale irregularity as the neuromuscular system explores alternative coordination patterns, while global scaling organisation at coarser time scales is maintained or reinforced through compensatory postural and mechanical adjustments [9,10]. Related nonlinear evidence from unstable-surface exercise, showing altered muscle activation variability and attentional demands under postural perturbation, provided further empirical grounding for this prediction [19,20,21]. 

## 2. Materials and Methods

### 2.1. Study Design

This repeated-measures cross-sectional study included two laboratory visits separated by 48–72 h. Participants were instructed to avoid moderate-to-vigorous physical activity for 24 h before testing and to abstain from alcohol, caffeine, tobacco, or stimulants for 4 h prior to each session [22]. All assessments were conducted under controlled environmental conditions at the same time of day.

### 2.2. Participants

Fifteen recreationally active participants (age: 31.4 ± 9.4 years; body mass: 72.4 ± 10.2 kg; height: 1.74 ± 0.1 m; BMI: 23.8 ± 2.7 kg·m^−2^; resting heart rate (HR): 66.24 ± 11.75 beats·min^−1^; 5 females) participated. Skinfold thicknesses at near-infrared spectroscopy (NIRS) placement sites were 9.8 ± 3.9 mm (*vastus lateralis*, VL) and 7.5 ± 2.9 mm (*gastrocnemius medialis*, GM). Inclusion criteria required at least 150 min·wk^−1^ of moderate-to-vigorous physical activity and familiarity with the exercise techniques; current or former competitive endurance runners were not systematically excluded, but training history and current competitive status were screened via the preliminary physical activity and health questionnaire, and all included participants were training recreationally at the time of testing. Exclusion criteria comprised diagnosed cardiovascular, pulmonary, or metabolic diseases, more than two cardiovascular risk factors, musculoskeletal or orthopaedic injuries, and subcutaneous tissue thickness > 15 mm at NIRS sites [23,24]. All participants provided written informed consent; the study was approved by the institutional ethics committee (Doc28-CE-UTAD-2022).

### 2.3. Procedures

#### 2.3.1. Preliminary Session

In the first session, participants completed an online physical activity and health questionnaire and the PAR-Q+ [25]. Anthropometric measurements (SECA 701 scale, SECA 220 stadiometer) and skinfold assessment (Slim Guide caliper, Creative Health Products, Ann Arbor, MI, USA) were obtained. Participants were familiarised with the protocol, exercise techniques, cadence target, and sensor attachment.

#### 2.3.2. Experimental Session

**Figure 1:**
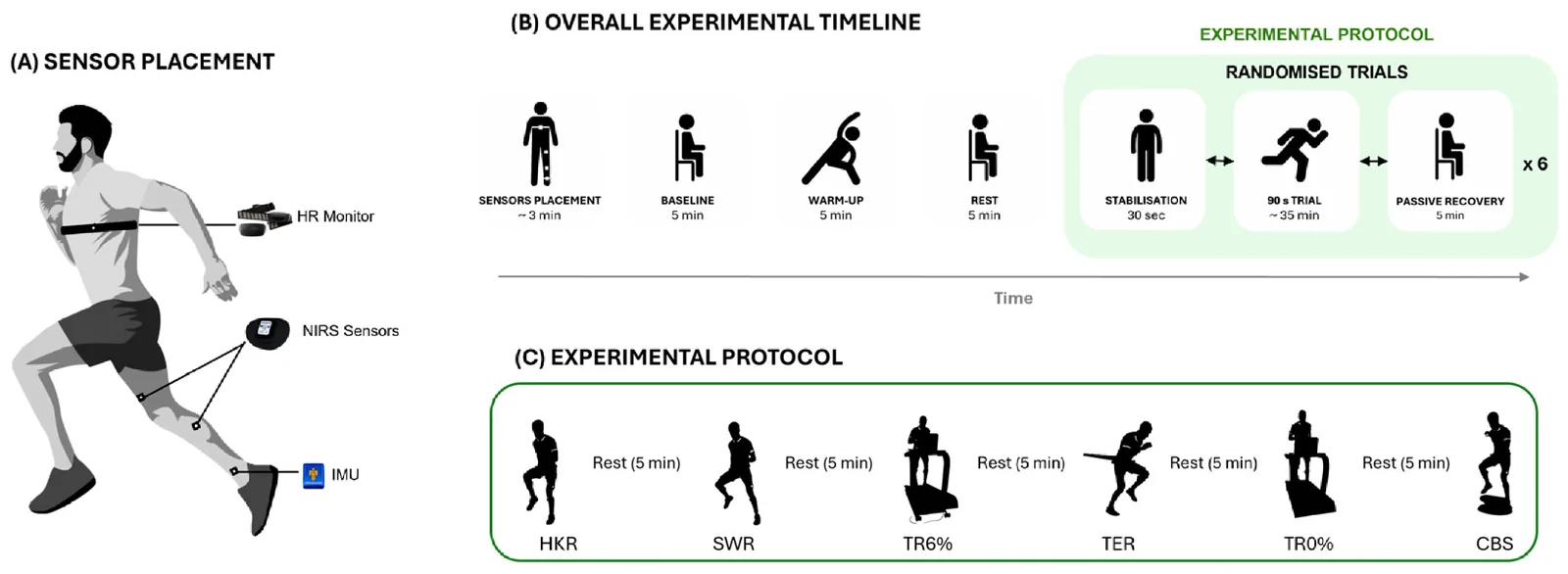
Experimental protocol: (**A**) Wearable sensors used for data acquisition (HR monitor, NIRS sensors, and IMU); (**B**) overview of the testing timeline; (**C**) sequence of the six randomised running variations, each performed for 90 s. Abbreviations: core-board skipping, CBS; heart rate, HR; high-knee running, HKR; inertial measurement unit, IMU; near-infrared spectroscopy, NIRS; shoulder-width running, SWR; tethered running, TER; treadmill running at 0% grade, TR0%; treadmill running at 6% grade, TR6%.

After a standardised warm-up (5 min light jogging followed by dynamic mobility exercises), participants completed six 90 s running conditions in randomised order, separated by 5 min of passive recovery. A 30 s quiet standing period preceded each condition to stabilise physiological variables (Figure 1).

#### 2.3.3. Exercise Variations

The six variations were (1) HKR, with feet shoulder-width apart, knees raised to 90° hip flexion, trunk maintained upright, and a crossed-arm pattern; (2) TER, with feet shoulder-width apart, knees raised to 90° hip flexion, trunk inclined forward, crossed-arm pattern, and elastic bands placed below the iliac crests; (3) SWR, with feet beyond shoulder-width, knees raised to 45°, arms following knee movement; (4) TR0%, treadmill running at 10.9 km·h^−1^ and 0% grade; (5) TR6%, treadmill running at 10.9 km·h^−1^ and 6% grade; and (6) CBS, running on an unstable platform (17 cm), knees raised to 45°, performed in front of a mirror to provide visual feedback and assist participants in maintaining the prescribed movement pattern. A metronome was used to maintain cadence at 130–150 bpm; a target elastic and floor markings standardised knee elevation and foot position.

### 2.4. Measurements and Instruments

HR was recorded continuously at 1 Hz using a Garmin HRM3-SS chest strap. SmO_2_ was sampled every 2 s (0.5 Hz) with two Moxy Monitor continuous-wave NIRS sensors (Fortiori Design LLC, Hutchinson, MN, USA) validated for exercise applications [24,26], placed on the VL and GM of the dominant leg following SENIAM recommendations (SENIAM, 2004) [27]. Sites were marked with permanent marker for reliability and sensors secured with Kinesio tape and light shields. Prior to each trial, real-time signal stability was confirmed over 30 s (variation < 5% per second); artefacts were excluded during post-processing. Both HR and SmO_2_ were streamed via ANT+ to a PC (SPRO, Hudl, Lincoln, NE, USA) and temporally aligned using synchronisation markers from the IMU (WIMU PRO, Hudl, Lincoln, NE, USA).

Acceleration data were collected at 1125 Hz using an IMeasureU Blue Trident IMU (Vicon Motion Systems, Oxford, UK) fixed 20 mm proximal to the medial malleolus [28,29,30]. Player load (PL) was computed as the per-sample magnitude of the rate of change in tri-axial acceleration, PL_t_ = √[(ax_t_ − ax_t−1_)^2^ + (ay_t_ − ay_t−1_)^2^ + (az_t_ − az_t−1_)^2^], following established inertial-load conventions [31]. For the PL-derived nonlinear analyses, exported PL time series were treated as the primary mechanical signal; four participant condition PL recordings were unavailable, yielding 86 valid PL series. Each 90 s series was harmonised to 128 Hz with anti-alias filtering, producing *N* = 11,520 samples per epoch.

#### 2.4.1. Data Processing and Derived Variables

The term “complexity” is used throughout this manuscript to encompass four conceptually distinct but empirically related properties of time series, each captured by a different metric. To ensure terminological precision and reproducibility, we define these explicitly: (a) signal regularity, the degree of pattern repetition within a single time scale, quantified by sample entropy (lower values = more regular); (b) multifractal richness, the breadth and shape of the singularity spectrum across multiple temporal scales, quantified by discrete wavelet leader (DWL) parameters (spectral width Δh, asymmetry L, dominant Hölder exponent h(0)); (c) global scaling structure, the long-range organisation of fluctuations across temporal scales, quantified by detrended fluctuation analysis exponent (DFA α) and Higuchi fractal dimension (Fd); and (d) ordinal irregularity, the diversity of rank-order patterns in local sequences, quantified by permutation entropy (PermEn). These dimensions are not interchangeable and may dissociate across conditions, as demonstrated in Section 3.

#### 2.4.2. Sample Entropy

SampEn was computed for each time series with embedding dimension m = 2 and tolerance r = 0.2 × SD, consistent with recommendations for biological data [32,33,34]. Because signals were sampled at different frequencies (HR 1 Hz, SmO_2_ 0.5 Hz, and PL 128 Hz after harmonisation), the delay was specified in samples at the native analysis resolution, rather than imposed as a single 100 ms delay across all signals. In practice, SampEn was computed with τ = 1 sample for each signal at its native analysis resolution, corresponding to 1 s for HR, 2 s for SmO_2_, and 7.8 ms for PL. The 90 s epoch therefore yielded markedly different series lengths across signals (approximately 90 samples for HR, 45 samples for SmO_2_, and 11,520 samples for PL). For HR and SmO_2_, these series lengths are below commonly recommended minima (typically ≥200–300 points) for stable, bias-free SampEn estimation with m = 2; consequently, HR and SmO_2_ SampEn values are reported and interpreted comparatively, as condition-specific indices of signal regularity under matched acquisition length, rather than as absolute, individually validated complexity estimates. The PL series, sampled at substantially higher frequency, provided a series length consistent with established SampEn recommendations. This distinction is reiterated in the Discussion and Limitations and additionally motivated the use of surrogate data testing to corroborate the multifractal PL findings, rather than relying on entropy estimates alone for the lower-frequency signals.

#### 2.4.3. Multifractal Discrete Wavelet Leader

Multifractal structure was characterised using the discrete wavelet leader (DWL) formalism, which quantifies the heterogeneity of local Hölder regularity across temporal scales [35,36,37]. Wavelet leaders were computed from the discrete wavelet transform using a Daubechies least-asymmetric wavelet filter (LA8). Positive and negative moment orders q emphasised large and small fluctuations, respectively. Moment orders ranged from q = −3 to +3 in increments of 0.5, and scaling exponents ζ(q) were estimated across dyadic scales j = 2–8 using weighted linear regressions of log_2_ S(q,j) on scale. This configuration was selected after parameter sensitivity analysis because it provided the best balance between scaling regression quality and conservative estimation of the spectrum tails. Each 90 s epoch contained 11,520 samples, and the coarsest retained scale contained approximately 45 wavelet leaders. The mean ζ(q) regression R^2^ across all participant condition series was 0.885. Parameters extracted from the singularity spectrum D(h) versus h are described in Supplementary Table S4 [38,39], and the detailed mathematical derivation of the estimator is provided in the Supplementary File S1. The high PL sampling density yielded 11,520 samples per 90 s epoch, but recording duration still limits the number of observations available at the coarsest temporal scales. We therefore constrained the scaling range to j = 2–8, evaluated regression quality, performed parameter sensitivity analyses, and used IAAFT and shuffled surrogates to assess whether empirical spectral width exceeded that expected from distributional or linear temporal structure alone. These procedures strengthen internal validity for the present acute protocol but do not establish equivalence with multifractal estimates obtained from longer recordings.

#### 2.4.4. Surrogate Data Validation of Multifractality

Surrogate data validation was used to test whether the observed multifractal width reflected genuine temporal organisation, rather than distributional structure alone. For each participant condition PL series, 99 iterative amplitude-adjusted Fourier transform (IAAFT) surrogates and 99 randomly shuffled surrogates were generated. IAAFT surrogates preserve the amplitude distribution and linear autocorrelation structure while disrupting nonlinear phase coupling, whereas shuffled surrogates preserve the marginal distribution but destroy temporal ordering [35,40]. Empirical multifractality was considered supported when the empirical spectral width Δh exceeded the 95th percentile of the corresponding surrogate distribution.

#### 2.4.5. Reduced Load Complexity Regime Analysis

To examine whether running variations differed in temporal complexity beyond physiological and mechanical load, four reduced dimensions were derived at the participant-by-condition level. Load magnitude was the average of standardised HR_mean_ and PL_mean_; load variability was the average of standardised HR_cv_ and PL_cv_. DFA α; Higuchi fractal dimension and permutation entropy were also reported as raw and standardised PL-derived nonlinear descriptors (Supplementary Table S5). DFA α was computed using first-order detrending over logarithmically spaced box sizes from 16 to N/4 samples (N/4 = 2880 for the 128 Hz PL series), Higuchi fractal dimension with kmax = 20, and permutation entropy with embedding dimension m = 5 and delay τ = 2 samples, normalised by log(m!). Because the association between DFA α and Higuchi fractal dimension was modest (r = 0.28), the reduced scaling complexity dimension was interpreted as an exploratory summary, rather than as evidence that both metrics were interchangeable.

The reduced regime analysis included 85 participant condition observations with matched HR and PL data, as one PL time series from TR0% PL could not be matched with a corresponding HR trace. Load magnitude and load variability were evaluated using participant-adjusted fixed-effects models with exercise condition and participant as factors. For the two temporal complexity dimensions, participant-adjusted models additionally included standardised HR_mean_ and PL_mean_ as covariates. Estimated marginal means and 95% confidence intervals were extracted for each condition from the corresponding reference grid. This approach isolates condition-specific temporal complexity differences after accounting for participant dependency and load magnitude while avoiding over-interpretation of the reduced dimensions as latent constructs.

#### 2.4.6. Sample Size Estimation and Justification

For the primary within-group comparisons across repeated measurements, an a priori power analysis was conducted using G*Power 3.1 [41], assuming a medium effect size (f = 0.25), two-tailed α = 0.05, power (1 − β) = 0.80, 6 repeated measurements, and a correlation among repeated measures of r = 0.5. Results indicated that a minimum sample size of *n* = 15 was required to detect an effect of this magnitude. This sample size was therefore adopted for the study. This a priori calculation targeted the primary within-condition physiological comparisons (HR, PL, SmO_2_) and does not fully account for the additional multiplicity introduced by the nonlinear and multifractal outcome families or for a sex-stratified analysis; with only 5 of 15 participants female, the study was not powered to detect sex differences, and condition effects are therefore reported and interpreted at the whole-sample level. Given this, together with the modest, mixed-sex sample size for a repeated-measures design spanning multiple nonlinear outcomes, the study is best regarded as a methodologically oriented pilot investigation, and the reported effects should be confirmed in a larger, sex-balanced sample before being generalised.

### 2.5. Statistical Analysis

Outliers and normality were screened using boxplots and Shapiro–Wilk tests. Linear mixed-effects models (LMMs) estimated the effects of exercise variation (fixed effect) on HR, PL, and SmO_2_ variables, with participant as a random intercept whenever model assumptions and convergence permitted. For the final PL-derived nonlinear analyses, condition effects were evaluated on all available participant condition series (*n* = 86) using participant-adjusted fixed-effects models (ordinary least squares with running condition and participant as factors; residual df = 66), which were used in place of the random intercept specification because the latter produced singular fits for several multifractal and PL-derived nonlinear outcomes. The reduced regime analysis used the subset of 85 observations with matched HR and PL data. Bonferroni-corrected pairwise comparisons were used for significant main effects. Effect sizes were Cohen’s d or dz for pairwise contrasts and partial η^2^ for main effects; conventional thresholds were applied (small: d = 0.2, ηp^2^ = 0.01; medium: d = 0.5, ηp^2^ = 0.06; large: d = 0.8, ηp^2^ = 0.14) [42]. Sample size (*n* = 15) was estimated a priori using G*Power (f = 0.25, α = 0.05, power = 0.80, 6 measurements, r = 0.5) [41]. Given the number of outcome families tested, the condition-level effects are interpreted as a coherent, hypothesis-driven set; the central nonlinear contrasts were pre-specified, and exact *p*-values are reported to support reader-side correction. Analyses were performed in R 4.5.1 (R Core Team, 2025) [43]. Significance was set at *p* ≤ 0.05. Data are presented as mean ± SD unless otherwise stated.

## 3. Results

### 3.1. Physiological and Mechanical Load Analysis

All variables showed a significant main effect of condition (largest *p* = 0.045, for SmO_2_ GM cv). Descriptive statistics and LMM results are summarised in Table 1.

Among HR metrics, CBS produced the lowest mean HR (123.63 ± 17.26 beats·min^−1^), significantly below HKR, TER, and TR6% (all *p* < 0.05; Table S1). HKR elicited the highest HR mean (144.66 ± 15.67 beats·min^−1^) and HR cv (11.67 ± 4.92%), differing significantly from SWR (*p* = 0.012, d = 0.825) and TR0% (*p* = 0.027, d = 1.168). Despite similar absolute mean values (~0.02) across conditions, HR SampEn differed significantly (*p* < 0.001, ηp^2^ = 0.07), with CBS showing systematically higher values than all other conditions (d = 0.74–1.16). Given the small absolute magnitude of these differences, HR SampEn should be interpreted primarily as evidence of condition-specific signal regularity under controlled cadence, rather than as a large physiological effect.

PL differences were substantial (PL mean: F = 102.78, ηp^2^ = 0.89). TR6% and TR0% reached the highest PL mean values (0.26 ± 0.03 and 0.25 ± 0.03, respectively), whereas CBS was lowest (0.10 ± 0.02; all pairwise *p* < 0.001; Table S2). HKR showed the highest PL mean among ground-based conditions (0.20 ± 0.04) but remained below treadmill conditions. PL SampEn was lowest during HKR (0.12 ± 0.04) and highest in CBS and TR6% (0.18 ± 0.03 and 0.18 ± 0.04), with dynamic conditions generally less complex than treadmill-based ones. PL cv was greatest during HKR (104.25 ± 13.65%) and SWR (101.31 ± 17.95%), with both exceeding treadmill conditions (all *p* < 0.001).

SmO_2_ patterns mirrored the intensity hierarchy. In both GM and VL, CBS yielded the highest mean SmO_2_ and HKR the lowest (Table 1 and Table S3). CBS GM mean (36.54 ± 22.30%) exceeded HKR (20.13 ± 4.18%; *p* = 0.004, d = 0.651), SWR, TER, and TR6% (all *p* < 0.05). In the VL, CBS VL mean (52.82 ± 8.09%) was significantly higher than HKR (41.94 ± 9.57%; *p* < 0.001, d = 1.796) and TR6% (*p* = 0.001, d = 1.061), while HKR was lower than SWR, TER, and TR0% (all *p* < 0.05). SmO_2_ cv was greatest during HKR in both muscles (GM cv: 91.50 ± 39.82%; VL cv: 40.77 ± 17.38%); CBS showed the lowest VL cv (12.77 ± 9.66%). SampEn of SmO_2_ remained low across conditions, but was significantly higher in CBS than all others in the GM (all d = 0.64–0.77), consistent with the metabolically quieter, unstable-surface task.

### 3.2. Multifractal Analysis

**Figure 2:**
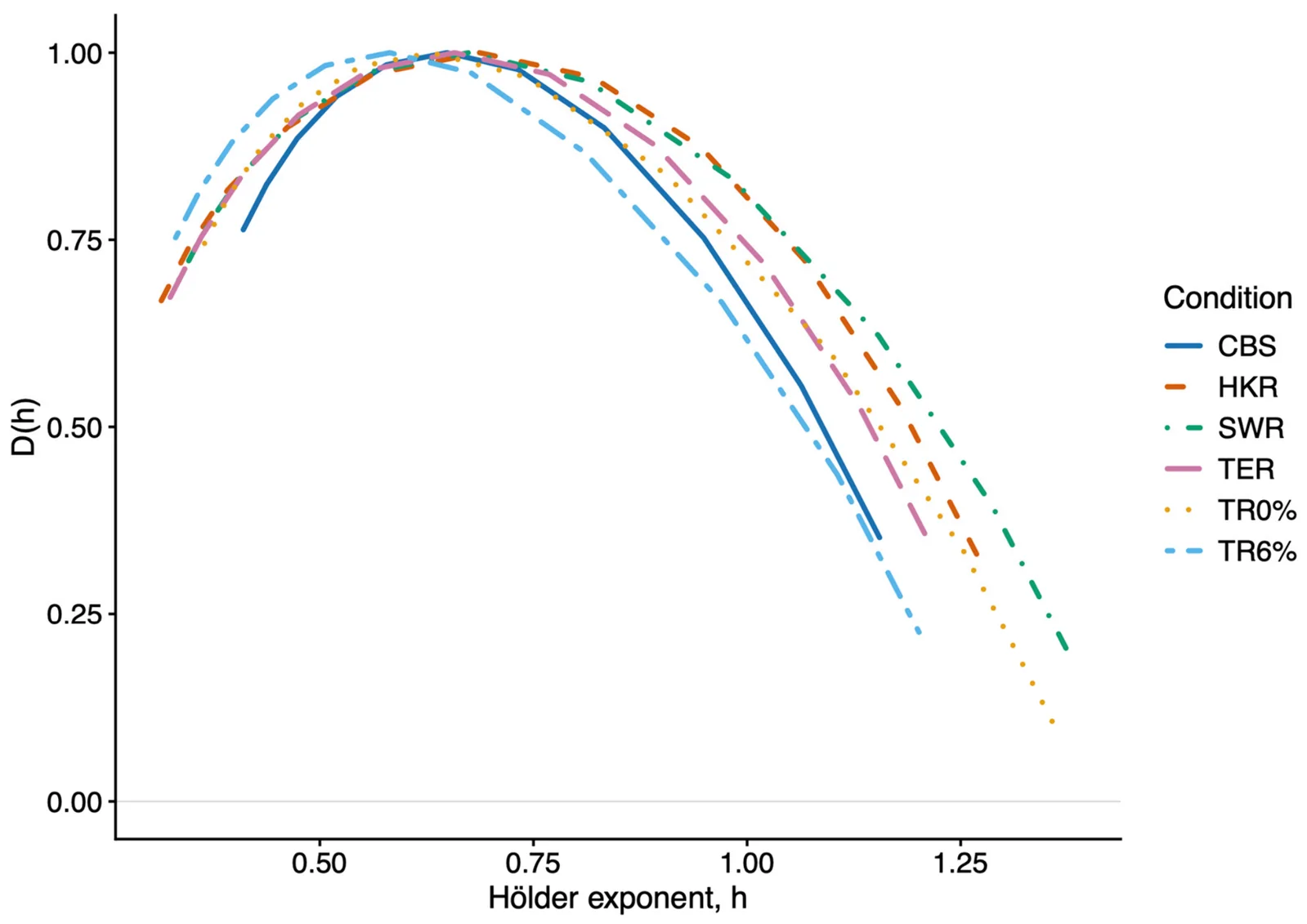
Group average wavelet leader multifractal spectra derived from PL series resampled to 128 Hz. Spectra were estimated using q = −3 to +3, dyadic scales j = 2–8, and LA8 wavelet leaders. Each 90 s epoch contained 11,520 samples; the coarsest retained scale contained approximately 45 leaders. Abbreviations: core-board skipping, CBS; high-knee running, HKR; shoulder-width running, SWR; tethered running, TER; treadmill running at 0%, TR0%; treadmill running at 6%, TR6%.

Figure 2 shows the group average multifractal spectra D(h) versus h estimated from the harmonised PL series using q = −3 to +3 and scales j = 2–8. The spectra were internally consistent with the tabulated DWL parameters. SWR, TR0%, and HKR showed numerically broader spectra and CBS the narrowest, although these spectral width (Δh) differences did not reach significance (Table 2); the significant condition effects were concentrated instead in dominant local regularity h(0), the most irregular local fluctuations hmin, and left-tail width L. The spectra also showed condition-specific shifts in dominant Hölder regularity and asymmetric tail extension, indicating that running variations differed not only in load magnitude, but also in the multiscale organisation of PL fluctuations.

**Table 2:** Descriptive statistics and participant-adjusted condition effects for wavelet leader multifractal spectrum parameters (Mean ± SD).

Variable	CBS (*n* = 15)	HKR (*n* = 14)	SWR (*n* = 14)	TER (*n* = 13)	TR0% (*n* = 15)	TR6% (*n* = 15)	df	F	*p*
h(0)	0.649 (0.044)	0.687 (0.049)	0.674 (0.062)	0.658 (0.056)	0.631 (0.043)	0.582 (0.053)	5, 66	9.73	<0.001
hmin	0.398 (0.046)	0.302 (0.030)	0.323 (0.038)	0.309 (0.035)	0.353 (0.044)	0.319 (0.046)	5, 66	17.62	<0.001
hmax	1.194 (0.250)	1.320 (0.206)	1.424 (0.388)	1.253 (0.272)	1.400 (0.430)	1.242 (0.401)	5, 66	0.96	0.447
Δh	0.796 (0.250)	1.019 (0.208)	1.102 (0.399)	0.944 (0.274)	1.048 (0.434)	0.923 (0.418)	5, 66	1.36	0.252
Dh(hmin)	0.732 (0.062)	0.636 (0.031)	0.665 (0.048)	0.634 (0.041)	0.714 (0.057)	0.722 (0.055)	5, 66	11.89	<0.001
Dh(hmax)	0.254 (0.349)	0.203 (0.317)	0.079 (0.371)	0.246 (0.343)	−0.001 (0.519)	0.123 (0.478)	5, 66	0.72	0.610
ΔDh	−0.478 (0.361)	−0.433 (0.313)	−0.587 (0.357)	−0.388 (0.365)	−0.715 (0.539)	−0.598 (0.464)	5, 66	0.98	0.437
L	0.251 (0.044)	0.385 (0.043)	0.352 (0.062)	0.349 (0.042)	0.278 (0.061)	0.263 (0.054)	5, 66	19.95	<0.001
R	0.546 (0.244)	0.633 (0.193)	0.750 (0.356)	0.596 (0.281)	0.770 (0.432)	0.660 (0.392)	5, 66	0.86	0.514
ΔS	0.295 (0.247)	0.248 (0.188)	0.399 (0.318)	0.247 (0.293)	0.492 (0.439)	0.397 (0.373)	5, 66	1.08	0.378

Notes: PL series were analysed at 128 Hz using q = −3 to +3 and dyadic scales j = 2–8. The coarsest retained scale contained 45 wavelet leaders. Pairwise comparisons for significant multifractal parameters are reported in Table 3. Abbreviations: core-board skipping, CBS; high-knee running, HKR; shoulder-width running, SWR; standard deviation, SD; tethered running, TER; treadmill running at 0%, TR0%; treadmill running at 6%, TR6%.

**Table 3:** Bonferroni-corrected post hoc pairwise comparisons for multifractal parameters with significant condition effects.

Parameter	Pairwise Comparison	Mean Difference [95% CI]	*p*	dz
h(0)	CBS × TR6%	0.067 [0.038, 0.096]	0.003	1.28
	HKR × TR6%	0.102 [0.056, 0.147]	0.005	1.28
	SWR × TR6%	0.097 [0.061, 0.134]	0.001	1.54
	TER × TR6%	0.070 [0.032, 0.108]	0.026	1.11
	TR0% × TR6%	0.049 [0.023, 0.075]	0.020	1.03
hmin	CBS × HKR	0.100 [0.070, 0.129]	<0.001	1.97
	CBS × SWR	0.074 [0.049, 0.099]	<0.001	1.72
	CBS × TER	0.089 [0.059, 0.119]	<0.001	1.80
	CBS × TR6%	0.079 [0.049, 0.110]	0.001	1.43
	HKR × TR0%	−0.052 [−0.079, −0.026]	0.014	−1.14
	TR0% × TR6%	0.034 [0.015, 0.053]	0.027	0.99
Dh(hmin)	CBS × HKR	0.101 [0.062, 0.140]	0.001	1.50
	CBS × SWR	0.064 [0.027, 0.102]	0.036	1.00
	CBS × TER	0.106 [0.060, 0.152]	0.004	1.40
	HKR × TR0%	−0.078 [−0.117, −0.039]	0.012	−1.15
	HKR × TR6%	−0.085 [−0.122, −0.048]	0.004	−1.33
	TER × TR0%	−0.075 [−0.118, −0.033]	0.035	−1.07
	TER × TR6%	−0.080 [−0.118, −0.042]	0.010	−1.27
L	CBS × HKR	−0.138 [−0.170, −0.106]	<0.001	−2.51
	CBS × SWR	−0.098 [−0.135, −0.061]	0.001	−1.52
	CBS × TER	−0.101 [−0.132, −0.071]	<0.001	−2.03
	HKR × TR0%	0.106 [0.060, 0.152]	0.004	1.33
	HKR × TR6%	0.124 [0.083, 0.166]	<0.001	1.72
	SWR × TR6%	0.086 [0.047, 0.125]	0.005	1.28
	TER × TR6%	0.077 [0.047, 0.107]	0.002	1.55

Abbreviations: core-board skipping, CBS; high-knee running, HKR; shoulder-width running, SWR; tethered running, TER; treadmill running at 0%, TR0%; treadmill running at 6%, TR6%.

**Figure 3:**
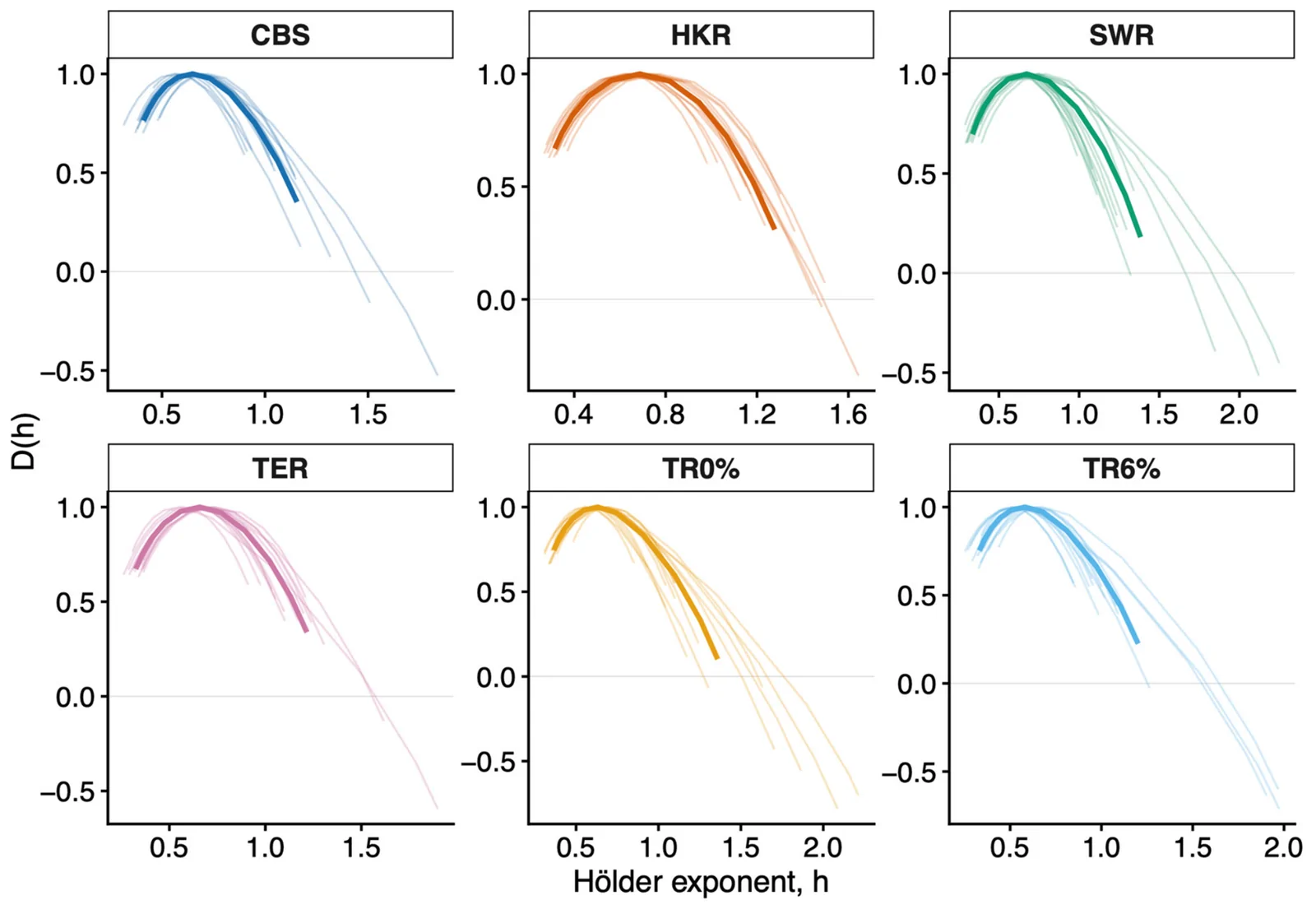
Individual wavelet leader multifractal spectra for each running condition. Thin lines represent participant condition spectra; thicker lines represent condition-level average spectra. Abbreviations: core-board skipping, CBS; high-knee running, HKR; shoulder-width running, SWR; tethered running, TER; treadmill running at 0%, TR0%; treadmill running at 6%, TR6%.

Individual spectra (Figure 3) revealed meaningful inter-individual dispersion around the condition-level spectra. CBS and HKR showed comparatively compact spectra in several participants, whereas SWR, TR0%, and TR6% displayed wider inter-individual variation in the right tail, which may indicate that inter-individual differences in motor-control strategies were more discernible in tasks with either greater locomotor constraint or treadmill-based mechanical regularity, although this inference is based on visual spectral dispersion, rather than a formal inter-individual variability statistic.

Among multifractal parameters (Table 2), *h*(0) differed significantly across conditions [F(5, 66) = 9.73, *p* < 0.001, ηp^2^ = 0.42]. The dominant Hölder exponent was above 0.5 in all conditions, with the highest values in HKR, SWR, and TER and the lowest value in TR6%. Thus, *h*(0) should be interpreted as a condition-specific shift in dominant local regularity, rather than as direct evidence of Hurst-type persistence or anti-persistence. *hmin* also differed significantly [F(5, 66) = 17.62, *p* < 0.001, ηp^2^ = 0.57], indicating condition-dependent differences in the most irregular local fluctuations.

*Dh(hmin)* differed significantly [F(5, 66) = 11.89, *p* < 0.001, ηp^2^ = 0.47], and the left-tail width *L* = *h*(0) − *hmin* showed the strongest condition effect [F(5, 66) = 19.95, *p* < 0.001, ηp^2^ = 0.60]. HKR, SWR, and TER generally displayed larger *L* than CBS and TR6%, consistent with greater left-tail extension and richer organisation of irregular local fluctuations in dynamic ground-based tasks. By contrast, *hmax*, Δ*h*, *Dh*(*hmax*), Δ*Dh*, *R*, and Δ*S* did not show significant condition effects. Surrogate data validation nevertheless confirmed that the observed spectral widths reflected genuine temporal multifractality: empirical Δ*h* exceeded the 95th percentile of IAAFT surrogates in 85 of 86 participant condition series and exceeded shuffled surrogates in all 86 series. At the condition level, empirical Δ*h* was consistently greater than both IAAFT and shuffled estimates (CBS: 0.796 vs. 0.483 and 0.189; HKR: 1.019 vs. 0.587 and 0.296; SWR: 1.102 vs. 0.575 and 0.273; TER: 0.944 vs. 0.510 and 0.280; TR0%: 1.048 vs. 0.434 and 0.231; TR6%: 0.923 vs. 0.411 and 0.228, respectively).

### 3.3. Temporal Complexity Regimes Beyond Load Magnitude

Before load adjustment, PL-derived nonlinear metrics differed significantly across conditions (Table S5): DFA α [F(5, 66) = 28.17, *p* < 0.001], Higuchi fractal dimension [F(5, 66) = 4.25, *p* = 0.002], and permutation entropy [F(5, 66) = 28.64, *p* < 0.001]. Raw values showed that DFA α was highest in SWR, HKR, CBS, and TER and lowest in TR6%, whereas permutation entropy was highest in CBS and lowest in the treadmill conditions. The correlation between DFA α and Higuchi fractal dimension was modest (r = 0.28), supporting their interpretation as complementary, exploratory descriptors of scaling structure, rather than interchangeable or validated indices of a single underlying construct.

**Figure 4:**
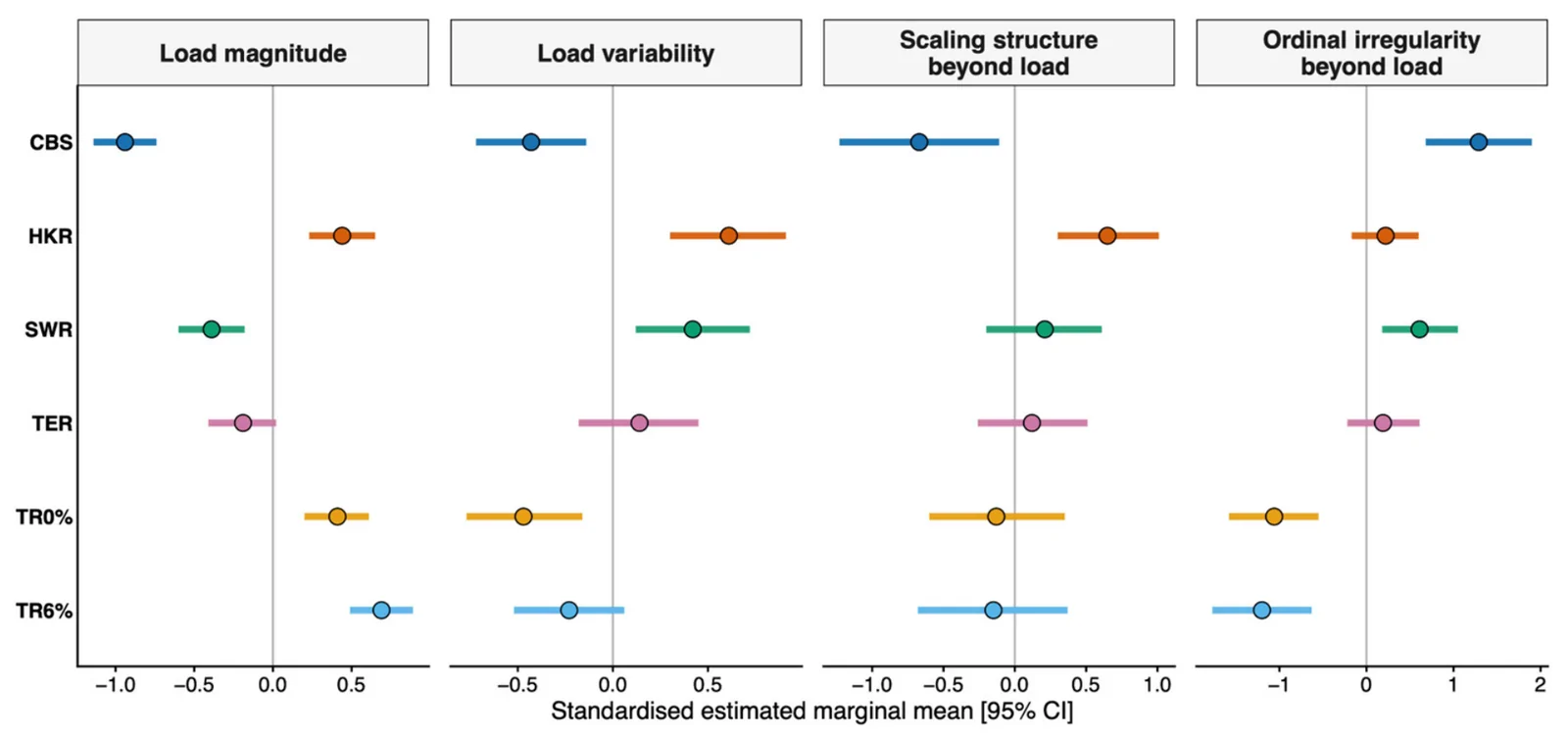
Load-adjusted temporal complexity regimes across running variations. Condition-level standardised effects are shown for load magnitude, load variability, scaling complexity beyond load, and ordinal irregularity beyond load. Values represent estimated marginal means from participant-adjusted fixed-effects models; complexity dimensions were additionally adjusted for HR _mean_ and PL _mean_. Error bars are 95% confidence intervals. Positive values indicate above-average expression; negative values indicate below-average expression. Load magnitude: F(5, 65) = 37.82, *p* < 0.001; load variability: F(5, 65) = 9.11, *p* < 0.001; scaling structure beyond load: F(5, 63) = 6.2, *p* < 0.001; ordinal irregularity beyond load: F(5, 63) = 6.50, *p* < 0.001. Abbreviations: core-board skipping, CBS; heart rate, HR; high-knee running, HKR; player load, PL; shoulder-width running, SWR; tethered running, TER; treadmill running at 0%, TR0%; treadmill running at 6%, TR6%.

Regime dimensions were then recomputed on the updated matched HR-PL dataset (85 participant condition observations; Table S6; Figure 4). Load magnitude remained highest in TR6% (0.69 [0.49, 0.89]) and HKR (0.44 [0.23, 0.65]) and lowest in CBS (−0.94 [−1.14, −0.74]). Load variability was greatest in HKR (0.61 [0.30, 0.91]) and SWR (0.42 [0.12, 0.72]) and lowest in TR0% (−0.47 [−0.77, −0.16]) and CBS (−0.43 [−0.72, −0.14]). Critically, the load-adjusted temporal complexity dimensions dissociated from load magnitude. After adjustment for HR_mean_ and PL_mean_, scaling complexity beyond load was highest in HKR (0.65 [0.30, 1.01]), while CBS was below the reference mean (−0.67 [−1.23, −0.11]) and treadmill conditions were close to or below zero (TR0%: −0.13 [−0.60, 0.35]; TR6%: −0.15 [−0.68, 0.37]). Because DFA and Higuchi captured only modestly overlapping information (r = 0.28), and because the absolute DFA α values were close to the uncorrelated boundary (approximately 0.41–0.54), this reduced dimension should be read as a compact, exploratory regime descriptor, rather than as evidence of strong long-range persistence or as a replacement for the raw metrics. Ordinal irregularity beyond load was highest in CBS (1.29 [0.68, 1.90]) and SWR (0.61 [0.18, 1.05]) and lowest in the treadmill conditions (TR0%: −1.06 [−1.58, −0.55]; TR6%: −1.20 [−1.77, −0.63]). Thus, CBS should not be interpreted as globally high in multiscale complexity; rather, it combined low load magnitude, high local ordinal irregularity, and a comparatively constrained multifractal spectrum. Overall, the running variations occupied distinct temporal complexity regimes, rather than representing interchangeable intensity manipulations.

## 4. Discussion

This study examined the influence of six running conditions on physiological intensity and movement complexity using linear and nonlinear analytical approaches. HR mean and variability differed between conditions; HR_SampEn_ also differed significantly, with CBS displaying a distinct autonomic modulation pattern despite small absolute differences. PL and SmO_2_ showed condition-specific differences across variability metrics, confirming distinct mechanical and local metabolic response profiles. Surrogate-validated multifractal analysis showed that PL fluctuations contained genuine temporal multifractality beyond distributional structure alone, and that running conditions differed mainly in dominant local regularity, the most irregular local fluctuations, and left-tail spectral extension. The load-adjusted regime analysis further showed that load magnitude and temporal complexity are partially dissociable.

### 4.1. Physiological and Mechanical Responses

HR_mean_ and HR_cv_ showed the most substantial physiological differences. HKR elicited the greatest cardiovascular demand, likely reflecting the high metabolic cost of sustained 90° hip flexion together with the restricted upper-body movement imposed by the crossed-arm position, which increases trunk stabilisation demands and muscular recruitment [44]. In contrast, CBS elicited the lowest cardiovascular demand, consistent with its lower external load.

Mechanical responses were also task-dependent. HKR showed the lowest PL_SampEn_, reflecting a highly repetitive loading pattern, whereas TR6% and CBS exhibited higher entropy, indicating greater stride-to-stride variability associated with unstable or less constrained movement conditions.

Although HR_SampEn_ differences were statistically significant, their absolute magnitude was very small (range ~0.00–0.01) and consistently localised to the CBS condition, which showed slightly higher values than the remaining conditions (d = 0.74–1.16). Given their very small absolute magnitude, these findings should be interpreted primarily as evidence of condition-specific heartbeat regularity under controlled cadence, rather than clinically meaningful physiological adaptations. Rather than reflecting unstable autonomic dynamics, this likely indicates that CBS, the lowest-intensity condition, experienced less cadence-driven suppression of inter-beat interval variability than conditions requiring greater cardiovascular engagement [45]. Externally imposed cadence constrains autonomic fluctuations during higher-intensity bouts [46,47], a floor effect that becomes more visible in the CBS context.

Mechanical responses were strongly task-dependent. HKR showed the lowest PL_SampEn_, reflecting a highly repetitive loading pattern, whereas TR6% and CBS exhibited higher entropy, consistent with increased stabilisation demands on inclined and unstable surfaces [48,49]. TR6% produced the highest PL_mean_, in line with the greater joint work demands of uphill running [50]. PL_CV_ was greatest in HKR and SWR, indicating more variable force generation relative to treadmill-based conditions, which displayed consistent, externally paced mechanical loading.

SmO_2 SampEn_ remained low across conditions in both VL and GM, consistent with the regularity imposed by rhythmical, externally paced exercise [51,52], but differed in CBS, reinforcing its distinct metabolic regime. Mean SmO_2_ was lowest during HKR, reflecting the greatest local mismatch between O_2_ delivery and O_2_ consumption driven by elevated recruitment at 90° hip flexion and incomplete reoxygenation between cycles, consistent with the GM’s dual role in propulsion and stabilisation [18,53,54,55].

### 4.2. Multiscale Structure of Movement Variability

Relative to the priori hypotheses, the multifractal predictions were only partially supported. The dominant Hölder exponent and the left-tail width L differed clearly between dynamic ground-based and treadmill conditions (H2, partially confirmed), but spectral width Δh did not differ significantly between conditions. CBS, which we had predicted would widen the spectrum and preserve or increase global scaling structure, instead showed the narrowest spectrum, low left-tail width L, and below-average scaling complexity beyond load, contrary to expectation. The final DWL analysis also did not support the earlier interpretation that all conditions exhibited h(0) < 0.5. Instead, the dominant Hölder exponent was above 0.5 across conditions and varied systematically, with HKR, SWR, and TER showing higher h(0), and TR6% the lowest value. This should be interpreted as a condition-specific shift in dominant local regularity, rather than as a direct Hurst-type index of long-range persistence [56]. The broader L values in HKR, SWR, and TER indicate greater left-tail extension and a wider range of irregular local fluctuations, consistent with richer rapid neuromuscular adjustments in dynamic ground-based tasks [57]. Treadmill conditions, especially TR6%, showed lower dominant local regularity and reduced left-tail extension, suggesting a more constrained organisation of rapid fluctuations under belt-driven locomotor constraints [58].

CBS merits specific attention because its nonlinear profile diverged across metrics. The DWL analysis showed the narrowest spectral width and lower L than the dynamic ground-based tasks, indicating constrained multifractal richness: the neuromuscular system apparently prioritised efficient, consistent control under postural perturbation [19,20,21]. However, the regenerated regime analysis showed that CBS was not above average in the reduced scaling complexity dimension after load adjustment, while ordinal irregularity beyond load was high. This dissociation is informative: DFA and Higuchi index global scaling properties, DWL characterises the heterogeneity of local fluctuation magnitudes across scales, and permutation entropy captures local rank-order diversity. CBS therefore appears to generate a movement signal that is locally irregular in ordinal structure, but comparatively homogeneous across scales, compatible with a stabilisation-dominant motor strategy under low physiological and mechanical load.

### 4.3. Implications for Exercise Selection and Individualised Prescription

The load-adjusted regime analysis provides a practical framework for exercise prescription beyond intensity [59]. HKR combined high-load magnitude and high-load variability with the clearest positive scaling complexity estimate beyond load within this reduced exploratory regime analysis, making it appropriate for well-conditioned individuals and athletes where high-intensity adaptive stimuli are the goal. TER offered a similar metabolic stimulus, but its load-adjusted scaling complexity estimate was close to the reference mean in the regenerated analysis; it may therefore be useful as a high-intensity resisted running stimulus without necessarily increasing global scaling complexity beyond load [60,61].

TR6% delivers the highest mechanical load and a strong cardiovascular stimulus and, because uphill running reduces peak vertical impact relative to high-speed level running, may represent a potential intensity progression stimulus worth exploring in return-to-running programmes for individuals with joint limitations; as the present sample comprised recreationally active adults, rather than rehabilitation patients, this application requires direct validation in the relevant clinical populations [50,62,63]. TR0% represents a controlled, cyclical mechanical stimulus with comparatively constrained residual complexity, which is useful when standardised, predictable mechanical delivery is the prescription goal [64].

CBS occupies a distinct niche: low physiological and mechanical load, high ordinal irregularity beyond load, and constrained multifractal richness. This profile may be worth exploring, in future studies with the relevant target groups, for clinical populations, deconditioned individuals, and fall-prevention programmes when the aim is to expose participants to local postural control variability without imposing high cardiovascular or mechanical load [65,66,67,68]. SWR presents an intermediate profile, with moderate load magnitude, elevated variability, and preserved ordinal irregularity, which may also warrant evaluation in general conditioning contexts.

The substantial inter-individual variability observed, particularly in treadmill-based conditions, confirms that standardised external conditions do not standardise internal response profiles [69]. Complexity-based metrics therefore offer a complementary monitoring dimension alongside traditional load indicators, with potential utility for tracking movement organisation, fatigue management, and readiness across both strength and conditioning and rehabilitation settings; because these data derive from a single acute testing session with short exercise bouts, this practical utility remains a hypothesis to be tested longitudinally, rather than an established application.

### 4.4. Limitations

Several limitations should be considered. Results are specific to recreationally active adults familiar with the exercise variations, and generalisation to clinical or athletic populations requires caution. The sample was also heterogeneous in age (31.4 ± 9.4 years) and anthropometric characteristics, spanning individuals closer to peak physiological performance capacity and others in earlier stages of performance decline; this variability, together with differences in subcutaneous tissue thickness and limb dimensions, may have contributed unmeasured variation in sensor placement and signal quality across participants. Given the modest, mixed-sex sample (*n* = 15; five females), the absence of a formal statistical comparison between sexes, and the resulting lack of a sex-specific subgroup analysis, the present findings should be regarded as pilot evidence for a novel multi-metric analytical approach, rather than as definitive population-level estimates; confirmation in a larger, more homogeneous, sex-balanced sample is required. The randomised condition order and 5 min passive recovery were intended to limit order and residual-fatigue effects, but the short 90 s bouts do not fully rule out such effects across six repeated conditions and may limit generalisability to longer, typical training durations. The 90 s epoch, while sufficient for acute physiological responses, may not fully characterise the multifractal properties of longer, steady-state efforts. Although surrogate validation supported genuine temporal multifractality, short exercise epochs can contain residual non-stationarity and drift; the multifractal parameters should therefore be interpreted together with scaling-quality diagnostics and sensitivity analyses. Single IMU placement on the ankle constrains assessment of proximal mechanical load. In addition, the final PL nonlinear dataset contained 86 valid participant condition series because four exported PL recordings were unavailable. Fixed cadence and duration enhanced comparability but may have suppressed natural movement variability. The reduced regime dimensions depend on the selected metrics and should be interpreted alongside the full LMM results and multifractal descriptors; load-adjusted models isolate complexity beyond HR_mean_ and PL_mean_ but do not imply causal independence between load and complexity.

A further uncontrolled confound concerns the CBS condition specifically: it was the only exercise performed in front of a mirror, introducing external visual feedback absent from all other conditions. Because visual feedback is commonly associated with reduced movement variability and increased attentional fixation, its presence may have attenuated, rather than amplified, some CBS-related variability estimates; however, this remains speculative. Future studies should include both mirror and no-mirror CBS conditions to disentangle the visual feedback and unstable-surface contributions.

Additionally, exercise intensity was indexed indirectly via HR and SmO_2_, rather than through measurement of oxygen uptake (VO_2_) or blood lactate. Although the fixed-cadence protocol facilitated comparability, future work should validate the intensity hierarchy with spirometry or lactate sampling to confirm that the observed physiological differences reflect genuine metabolic gradation, rather than cardiorespiratory regulation artefacts.

## 5. Conclusions

Distinct running variations produce specific physiological, mechanical, and movement complexity profiles that are not interchangeable and cannot be fully captured by linear metrics alone. Consequently, the observed task-specific responses provide a rationale for tailoring exercise selection according to the intended physiological, mechanical, and rehabilitation objectives. High-knee running emerged as a potent high-intensity stimulus combining high mechanical load and scaling complexity, whereas TR6% may offer a controlled approach to increasing mechanical and cardiovascular demand with reduced impact constraints, a potential application for exercise progression and return-to-running contexts that warrants direct testing in the relevant populations. Conversely, core-board skipping offered a low-load yet irregular stimulus that may hold promise for early-stage rehabilitation and postural control training pending confirmation in such populations, while TR0% enabled highly standardised mechanical exposure.

## Figures and Tables

**Table 1:** Descriptive statistics and linear mixed models ANOVA for HR, PL, and SmO_2_ variables (mean ± SD).

Variables	CBS (a)	HKR (b)	SWR (c)	TER (d)	TR0% (e)	TR6% (f)	df	F	*p*
HR_SampEn_ (a.u.)	0.02 (0.01) ^b–f^	0.02 (0.0)	0.02 (0.0)	0.02 (0.0)	0.02 (0.0)	0.02 (0.0)	5, 64.13	7.10	<0.001
HR_mean_ (beats·min^−1^)	123.63 (17.26) ^b,d,f^	144.66 (15.67) ^c,e^	132.54 (15.89)	136.94 (12.53)	130.67 (8.03)	139.49 (15.27)	5, 64.92	8.73	<0.001
HR_cv_ (%)	8.22 (2.43) ^b,d,f^	11.67 (4.92) ^e^	10.19 (2.68)	11.66 (3.44) ^e^	8.51 (1.82)	10.93 (1.87)	5, 63.26	5.12	<0.001
PL_SampEn_ (a.u.)	0.18 (0.03) ^b,c,d^	0.12 (0.04) ^e,f^	0.14 (0.04) ^f^	0.13 (0.03) ^e,f^	0.17 (0.02)	0.18 (0.04)	5, 61.89	8.31	<0.001
PL_mean_ (a.u.)	0.10 (0.02) ^b–f^	0.20 (0.04) ^c–f^	0.15 (0.04) ^e,f^	0.15 (0.02) ^e,f^	0.25 (0.03)	0.26 (0.03)	5, 61.46	102.78	<0.001
PL_cv_ (%)	86.33 (6.69) ^b,c^	104.25 (13.65) ^e,f^	101.31 (17.95) ^e,f^	92.34 (13.09) ^f^	82.76 (11.07)	79.25 (9.37)	5, 65.62	12.46	<0.001
SmO_2_ GM_SampEn_ (a.u.)	0.02 (0.01) ^b–f^	0.01 (0.0)	0.01 (0.0)	0.01 (0.0)	0.01 (0.0)	0.01 (0.0)	5, 53.53	6.29	<0.001
SmO_2_ GM_mean_ (%)	36.54 (22.30) ^b,c,d,f^	20.13 (4.18)	23.81 (7.09)	24.39 (8.36)	27.03 (6.91)	23.23 (8.03)	5, 59.50	4.07	0.003
SmO_2_ GM_cv_ (%)	61.14 (52.54)	91.50 (39.82)	69.50 (30.89)	85.48 (39.43)	70.48 (29.79)	74.92 (23.99)	5, 59.85	2.44	0.045
SmO_2_ VL_SampEn_ (a.u.)	0.01 (0.0) ^b^	0.01 (0.0) ^d–f^	0.01 (0.0)	0.01 (0.0)	0.01 (0.0)	0.01 (0.01)	5, 49.73	6.57	<0.001
SmO_2_ VL_mean_ (%)	52.82 (8.09) ^b,f^	41.94 (9.57) ^c–e^	47.81 (10.59)	48.99 (13.11)	48.49 (5.09)	43.61 (11.89)	5, 58.14	7.21	<0.001
SmO_2_ VL_cv_ (%)	12.77 (9.66) ^b,f^	40.77 (17.38) ^c–f^	21.72 (11.02) ^f^	20.07 (11.43) ^f^	16.63 (9.96) ^f^	32.11 (18.98)	5, 61.95	25.07	<0.001

Notes: Superscripts indicate significant difference (*p* ≤ 0.05) vs. the labelled condition (a = CBS, b = HKR, c = SWR, d = TER, e = TR0%, f = TR6%). b–f indicate significant difference vs. all five other conditions. Abbreviations: coefficient of variation, CV; core-board skipping, CBS; *Gastrocnemius medialis,* GM; heart rate, HR; high-knee running, HKR; linear mixed-effects models, LMM; muscle oxygenation, SmO_2_; player load, PL; sample entropy, SampEn; shoulder-width running, SWR; tethered running, TER; treadmill running at 0% grade, TR0%; treadmill running at 6% grade, TR6%; *Vastus lateralis*, VL.

## Data Availability

The data presented in this study are available on reasonable request from the corresponding author.

## Supplementary Materials

The following supporting information can be downloaded at: https://www.mdpi.com/article/10.3390/s26185881/s1, File S1: Multifractal Discrete Wavelet Leader (DWL); Table S1: Bonferroni-Corrected Pairwise Comparisons for Significant Differences in Heart Rate Variables; Table S2: Bonferroni-Corrected Pairwise Comparisons for Significant Differences in Player Load Variables; Table S3: Bonferroni-Corrected Pairwise Comparisons for Significant Differences in Muscle Oxygenation Variables; Table S4: Detailed description of parameters extracted from the multifractal spectrum [68,69]; Table S5: Raw and standardised individual nonlinear PL-derived metrics by running condition and global participant-adjusted condition effects; Table S6: Reduced load-complexity regime dimensions: model-estimated condition effects; Supplementary Note: Mathematical Derivation of the Discrete Wavelet Leader Estimator [32,33].

## Abbreviations

The following abbreviations are used in this manuscript:

BMI	Body Mass Index
CBS	Core-board Skipping
CV	Coefficient of Variation
DFA	Detrended Fluctuation Analysis
DWL	Discrete Wavelet Leader
GM	*Gastrocnemius Medialis*
HKR	High-knee Running
HR	Heart Rate
IAAFT	Iterative Amplitude-Adjusted Fourier Transform
IMU	Inertial Measurement Unit
LMM	Linear Mixed-effects Model
NIRS	Near-Infrared Spectroscopy
PL	Player Load
SampEn	Sample Entropy
SD	Standard Deviation
SmO_2_	Muscle Oxygenation
SWR	Shoulder-width Running
TER	Tethered Running
TR0%	Treadmill Running at 0% Grade
TR6%	Treadmill Running at 6% Grade
VL	*Vastus Lateralis*

## References

[1] Bartlett R., Wheat J., Robins M. (2007). Is movement variability important for sports biomechanists?. Sports Biomech..

[2] Cowin J., Nimphius S., Fell J., Culhane P., Schmidt M. (2022). A Proposed Framework to Describe Movement Variability within Sporting Tasks: A Scoping Review. Sports Med.-Open.

[3] Silva P., Duarte R., Sampaio J., Aguiar P., Davids K., Araújo D., Garganta J. (2014). Field dimension and skill level constrain team tactical behaviours in small-sided and conditioned games in football. J. Sports Sci..

[4] Di Bacco V., Gage W. (2024). Gait variability, fractal dynamics, and statistical regularity of treadmill and overground walking recorded with a smartphone. Gait Posture.

[5] Padulo J., Ayalon M., Barbieri F., Di Capua R., Doria C., Ardigò L., Dello Iacono A. (2023). Effects of Gradient and Speed on Uphill Running Gait Variability. Sports Health.

[6] Hunter B., Karsten B., Greenhalgh A., Burnley M., Muniz-Pumares D. (2023). The Application of non-linear methods to quantify changes to movement dynamics during running: A scoping review. J. Sports Sci..

[7] Yentes J., Raffalt P. (2021). Entropy Analysis in Gait Research: Methodological Considerations and Recommendations. Ann. Biomed. Eng..

[8] Kelty-Stephen D., Lee J., Cole K., Shields R., Mangalam M. (2023). Multifractal Nonlinearity Moderates Feedforward and Feedback Responses to Suprapostural Perturbations. Percept. Mot. Ski..

[9] Kantelhardt J., Zschiegner S., Koscielny-Bunde E., Havlin S., Bunde A., Stanley H. (2002). Multifractal detrended fluctuation analysis of nonstationary time series. Physica A Stat. Mech. Its Appl..

[10] Kelty-Stephen D., Palatinus K., Saltzman E., Dixon J. (2013). A Tutorial on Multifractality, Cascades, and Interactivity for Empirical Time Series in Ecological Science. Ecol. Psychol..

[11] Mendez M., Bianchi A., Recker F., Strizek B., Murguía J., Reali P., Jimenez-Cruz J. (2024). Multifractal analysis of heart rate variability in pregnancy during sleep. Front. Cardiovasc. Med..

[12] Zorick T., Landers J., Leuchter A., Mandelkern M. (2020). EEG multifractal analysis correlates with cognitive testing scores and clinical staging in mild cognitive impairment. J. Clin. Neurosci. Off. J. Neurosurg. Soc. Australas..

[13] Babault N., Hitier M., Paizis C., Vieira D. (2023). Exploring Acute Changes in Hamstring EMG after Warm-up and Stretching Using a Multifractal Analysis. Med. Sci. Sports Exerc..

[14] Babault N., Rodot G., Cometti C., Vieira D. (2024). Elite women’s soccer match demand can be described using complexity-based analyses and multifractals. Chaos Solitons Fractals.

[15] Cruz I., Sampaio J. (2020). Multifractal Analysis of Movement Behavior in Association Football. Symmetry.

[16] Ramos Y., de Arruda H., Copelli M., Camelo-Neto G., Carelli P. (2026). Multifractal human signals at the edge of life reveal a heart-brain anti-correlation. arXiv.

[17] Exel J., Mateus N., Gonçalves B., Abrantes C., Calleja-González J., Sampaio J. (2019). Entropy Measures Can Add Novel Information to Reveal How Runners’ Heart Rate and Speed Are Regulated by Different Environments. Front. Psychol..

[18] Gómez-Carmona C., Bastida-Castillo A., Rojas-Valverde D., de la Cruz Sánchez E., García-Rubio J., Ibáñez S., Pino-Ortega J. (2020). Lower-limb Dynamics of Muscle Oxygen Saturation During the Back-squat Exercise: Effects of Training Load and Effort Level. J. Strength Cond. Res..

[19] Khajooei M., Quarmby A., Mayer F., Engel T. (2024). Biomechanical feedback and feedforward responses during perturbed running in asymptomatic individuals. Front. Sports Act. Living.

[20] Mademli L., Mavridi D., Bohm S., Patikas D., Santuz A., Arampatzis A. (2021). Standing on unstable surface challenges postural control of tracking tasks and modulates neuromuscular adjustments specific to task complexity. Sci. Rep..

[21] Munoz-Martel V., Santuz A., Ekizos A., Arampatzis A. (2019). Neuromuscular organisation and robustness of postural control in the presence of perturbations. Sci. Rep..

[22] Gibson A., Wagner D., Heyward V. (2024). Advanced Fitness Assessment and Exercise Prescription.

[23] Barstow T. (2019). Understanding near infrared spectroscopy and its application to skeletal muscle research. J. Appl. Physiol..

[24] Feldmann A., Schmitz R., Erlacher D. (2019). Near-infrared spectroscopy-derived muscle oxygen saturation on a 0% to 100% scale: Reliability and validity of the Moxy Monitor. J. Biomed. Opt..

[25] Warburton D., Jamnik V., Bredin S., Shephard R., Gledhill N. (2019). The 2020 physical activity readiness questionnaire for everyone (PAR-Q+) and electronic physical activity readiness medical examination (ePARmed-X+): 2020 PAR-Q+. Health Fit. J. Can..

[26] Crum E., O’Connor W., Van Loo L., Valckx M., Stannard S. (2017). Validity and reliability of the Moxy oxygen monitor during incremental cycling exercise. Eur. J. Sport Sci..

[27] Hermens H.J., Freriks B., Disselhorst-Klug C., Rau G. (2000). Development of recommendations for SEMG sensors and sensor placement procedures. J. Electromyogr. Kinesiol..

[28] Rice H., Saunders S., McGuire S., O’Leary T., Izard R. (2018). Estimates of Tibial Shock Magnitude in Men and Women at the Start and End of a Military Drill Training Program. Mil. Med..

[29] Sheerin K., Besier T., Reid D. (2020). The influence of running velocity on resultant tibial acceleration in runners. Sports Biomech..

[30] Sheerin K., Besier T., Reid D., Hume P. (2018). The one-week and six-month reliability and variability of three-dimensional tibial acceleration in runners. Sports Biomech..

[31] Gómez-Carmona C., Bastida-Castillo A., González-Custodio A., Olcina G., Pino-Ortega J. (2020). Using an Inertial Device (WIMU PRO) to Quantify Neuromuscular Load in Running: Reliability, Convergent Validity, and Influence of Type of Surface and Device Location. J. Strength Cond. Res..

[32] Richman J., Moorman J. (2000). Physiological time-series analysis using approximate entropy and sample entropy. Am. J. Physiol. Heart Circ. Physiol..

[33] Stergiou N., Buzzi H., Kurz M., Heidel J., Stergiou N. (2004). Nonlinear tools in human movement. Innovative Analyses of Human Movement: Analytical Tools for Human Movement Research.

[34] Yentes J., Entropy, Stergiou N. (2017). Nonlinear Analysis for Human Movement Variability.

[35] Ihlen E., Vereijken B. (2013). Multifractal formalisms of human behavior. Hum. Mov. Sci..

[36] Jaffard S., Lashermes B., Abry P. (2006). Wavelet leaders in multifractal analysis. Wavelet Analysis and Applications.

[37] Wendt H., Abry P., Jaffard S. (2007). Bootstrap for Empirical Multifractal Analysis. IEEE Signal Process. Mag..

[38] Munro M., Ord A., Hobbs B. (2018). Spatial organization of gold and alteration mineralogy in hydrothermal systems: Wavelet analysis of drillcore from Sunrise Dam Gold Mine, Western Australia. Geol. Soc. Lond. Spec. Publ..

[39] Zhang X., Liu H., Zhao Y., Zhang X. (2019). Multifractal detrended fluctuation analysis on air traffic flow time series: A single airport case. Phys. A Stat. Mech. Its Appl..

[40] Schreiber T., Schmitz A. (2000). Surrogate time series. Physica D. Nonlinear Phenom..

[41] Faul F., Erdfelder E., Lang A., Buchner A. (2007). G*Power 3: A flexible statistical power analysis program for the social, behavioral, and biomedical sciences. Behav. Res. Methods.

[42] Cohen J. (2013). Statistical Power Analysis for the Behavioral Sciences.

[43] Team R.C. (2025). R: A Language and Environment for Statistical Computing, 4.5.1.

[44] Listiwikono E., Supono S., Mustain A., Setiawan W., Mursidi A. (2022). Effect of high knee running and high knee bounce skips exercises on running speed of 100 meters of athletic extracurricular participants in senior high school. Linguist. Cult. Rev..

[45] Stergiou N., Decker L. (2011). Human movement variability, nonlinear dynamics, and pathology: Is there a connection?. Hum. Mov. Sci..

[46] Caballero C., Davids K., Heller B., Wheat J., Moreno F. (2019). Movement variability emerges in gait as adaptation to task constraints in dynamic environments. Gait Posture.

[47] Nurrosyidah A., Mahananto F., Er M., Igasaki T., Yamakawa T. (2019). Heart Rate Variability Analysis by Multiscale Entropy for Autonomic Nervous System Identification. Procedia Comput. Sci..

[48] Claußen L., Braun C. (2023). Challenge Not Only to the Muscles—Surface Instability Shifts Attentional Demands in Young and Older Adults While Performing Resistance Exercise. J. Cogn. Enhanc..

[49] Lehmann T., Visser A., Havers T., Büchel D., Baumeister J. (2022). Surface Instability Modulates Cortical Information Processing In Multi-Joint Compound Movement. Med. Sci. Sports Exerc..

[50] Vernillo G., Giandolini M., Edwards W., Morin J., Samozino P., Horvais N., Millet G. (2017). Biomechanics and Physiology of Uphill and Downhill Running. Sports Med..

[51] Molinari F., Acharya R., Martis R., De Luca R., Petraroli G., Liboni W. (2013). Entropy analysis of muscular near-infrared spectroscopy (NIRS) signals during exercise programme of type 2 diabetic patients: Quantitative assessment of muscle metabolic pattern. Comput. Methods Programs Biomed..

[52] Montull L., Balagué N., Petelczyc M., Marszalek K., Vázquez P. (2025). Time-variability of muscle oxygen saturation during graded maximal exercise. Eur. J. Appl. Physiol..

[53] Davis P., Yakel J., Anderson D. (2020). Muscle Oxygen Demands of the Vastus Lateralis in Back and Front Squats. Int. J. Exerc. Sci..

[54] Hamner S., Seth A., Delp S. (2010). Muscle contributions to propulsion and support during running. J. Biomech..

[55] Spencer M., Murias J., Paterson D. (2012). Characterizing the profile of muscle deoxygenation during ramp incremental exercise in young men. Eur. J. Appl. Physiol..

[56] Ihlen E. (2012). Introduction to Multifractal Detrended Fluctuation Analysis in Matlab. Front. Physiol..

[57] Delignieres D., Marmelat V. (2012). Fractal fluctuations and complexity: Current debates and future challenges. Crit. Rev. BioMed Eng..

[58] Dingwell J., Cusumano J. (2000). Nonlinear time series analysis of normal and pathological human walking. Chaos.

[59] ACSM (2021). ACSM’s Guidelines for Exercise Testing and Prescription.

[60] McMorrow B., Ditroilo M., Egan B. (2019). Effect of Heavy Resisted Sled Sprint Training During the Competitive Season on Sprint and Change-of-Direction Performance in Professional Soccer Players. Int. J. Sports Physiol. Perform..

[61] Petrakos G., Morin J., Egan B. (2016). Resisted Sled Sprint Training to Improve Sprint Performance: A Systematic Review. Sports Med..

[62] Telhan G., Franz J., Dicharry J., Wilder R., Riley P., Kerrigan D. (2010). Lower limb joint kinetics during moderately sloped running. J. Athl. Train..

[63] Pamboris G., Nelson A., Williams L., Fong H., Senatore S., Lyons S., Peel S., Sandiford T., Powell D. (2025). Inclined distance running at iso-efficient speeds: Effect on joint work. J. Sports Sci..

[64] DeJong Lempke A., Audet A., Wasserman M., Melvin A., Soldes K., Heithoff E., Shah S., Kozloff K., Lepley A. (2025). Biomechanical differences and variability during sustained motorized treadmill running versus outdoor overground running using wearable sensors. J. Biomech..

[65] Behm D., Colado J. (2012). The effectiveness of resistance training using unstable surfaces and devices for rehabilitation. Int. J. Sports Phys. Ther..

[66] Behm D., Colado J., Colado J. (2013). Instability resistance training across the exercise continuum. Sports Health.

[67] Hamed A., Bohm S., Mersmann F., Arampatzis A. (2018). Exercises of dynamic stability under unstable conditions increase muscle strength and balance ability in the elderly. Scand. J. Med. Sci. Sports.

[68] Williams D., Murray N., Powell D. (2016). Athletes who train on unstable compared to stable surfaces exhibit unique postural control strategies in response to balance perturbations. J. Sport Health Sci..

[69] Hecksteden A., Pitsch W., Rosenberger F., Meyer T. (2018). Repeated testing for the assessment of individual response to exercise training. J. Appl. Physiol..

