# Supplementary material for: Running Constraints Shape Multifractal Movement Complexity Beyond Physiological and Mechanical Load: A Pilot Study
Source: Sensors (Basel). 2026 Sep 17;26(18):5881. doi: 10.3390/s26185881 (PMC13611035; doi:10.3390/s26185881)
Supplement: Supplementary file 1 [file sensors-26-05881-s001.zip › Multifractal Discrete Wavelet Leader (DWL) File S1.pdf]

File S1. Multifractal Discrete Wavelet Leader (DWL)

Multifractal analysis was employed to characterize the temporal organization of movement variability across multiple time scales, which cannot be captured by traditional linear or monofractal measures (Ihlen, 2012) The DWL is an analytical technique rooted in signal processing that provides a unified approach to the multifractal analysis of complex signals (Jaffard et al., 2006).

Essentially, the DWL aims to characterize the temporal fluctuations of the local singularity in a signal $x[t]$ or function$f(t)$. This is achieved by quantifying the size of sets of points in $\mathbb{R}^d$ that share the same local singularity h. Local singularities are measured by pointwise exponents, the most common of which is the Hölder exponent $h_f(t_0)$, defined as follows:

The function f belongs to the class $C^\alpha(t_0)$ with $\alpha \geq 0$, if there exists a polynomial P_{t_0} of degree less than α and a constant $C > 0$ such that:

$$|f(t_0 + a) - P_{t_0}(t_0 + a)| \leq C|a|^\alpha, \quad as\ |a| \to 0$$

The Hölder exponent $h(t_0)$ is the largest α for which $f \in C^\alpha(t_0)$, i.e.,

$$h(t_0) = sup\{\alpha : f \in C^\alpha(t_0)\} \geq 0$$

Essentially, larger values of $h(t_0)$ indicate that f is smoother around t_0, whereas values closer to zero correspond to more irregular local behavior. The Wavelet leaders have been theoretically proven to be the only multiscale measure capable of accurately characterizing the Hölder exponent (Jaffard et al., 2016).

Signal decomposition via Discrete Wavelet Transform

To describe the characteristics of local singularities, DWL analysis relies on the Discrete Wavelet Transform (DWT) of the signal $X = \{x_k, k \in \mathbb{Z}\}$, defined as:

$$d_x(j,k) = \int X(t) 2^{-j} \psi_0(2^{-j}t - k)dt$$

where $\psi(t)$ is the "mother" wavelet with compact support, t is the time variable, j is the dyadic scale parameter, and k is the translation parameter. According to Ihlen and Vereijken (2013), the DWT computes the scale-dependent measure $d_x(j,k)$ through recursive high-pass and low-pass filtering of the signal X. Specifically, the coefficient at scale $j = 1$ is obtained by convolving the signal with a high-pass filter followed by subsampling by 2. This process is repeated for higher scales $j = 2,3, \ldots$ having the sample size at each level while doubling the frequency band analyzed.

Wavelet Leaders Calculation

Wavelet leaders $L_x(j,k)$ are defined as the local maxima of the wavelet coefficients $d_x(j,k)$ over three consecutive dyadic intervals $\lambda_{j,k}$ at scale j and all finer scales $j' < j$:

$$L_x(j,k) \ = sup_{\lambda' \in 3\lambda} \ |d_x(j,k)|$$

In this context, where sup refers to the supremum, corresponding to the local maximum of the wavelet coefficients $d_x(j',k)$ in the neighbourhood.

Hölder exponents and multifractal spectrum estimation

The size and variability of the sets of points where the pointwise singularity exponent takes a specific value h are formalized by the Hausdorff dimension $D(h)$ (Fuss et al., 2012).

The multifractal spectrum $D(h)$ is obtained indirectly through the Legendre transform of the statistical moments of order q of the local scale-dependent measure $L_X(j,k)$. The structure function $S(j,q)$ and scaling exponents $\zeta(q)$ are defined as:

$$S(q,j) = \frac{1}{n_j} \sum_{k=1} |L_x(j,k)|^q,$$

$$\zeta(q) = \lim_{j \to 0} inf \left(\frac{\log S\ (j,q)}{\log 2^{-j}} \right),$$

where q the statistical order, and n_j the number of leaders in scale j. The scale exponent $\zeta(q)$ is determined by linear regression of $log\ \ S(q,j)$ as a function of $\log 2^j$.The spectrum $D(h)$ was then obtained by Legendre transform:

$$D(h) = inf_{\{q \neq 0\}}(1 + qh - \zeta(q)).$$

As noted by Ihlen and Vereijken (2013), $\zeta(q)$ is assumed to be a convex function of q, with its slope, the singularity exponent h_q, decreasing monotonically with q. Positive q-orders emphasize large fluctuations in the signal, while negative q-orders highlight small fluctuations, corresponding to the left and right tails of the singularity spectrum, respectively.

In the special case of monofractal noise-like time series, $\zeta(q)$ is linear in q, implying that h_q and $D(h)$ are constants independent of q, resulting in a very narrow singularity spectrum.
