# Supplementary material for: Running Constraints Shape Multifractal Movement Complexity Beyond Physiological and Mechanical Load: A Pilot Study
Source: Sensors (Basel). 2026 Sep 17;26(18):5881. doi: 10.3390/s26185881 (PMC13611035; doi:10.3390/s26185881)
Supplement: Supplementary file 1 [file sensors-26-05881-s001.zip › Supplementary Note.pdf]

Supplementary Note: Mathematical Derivation of the Discrete Wavelet Leader Estimator

The Discrete Wavelet Leader (DWL) characterises temporal fluctuations of local singularities in a signal $x[t]$ by quantifying the size of sets of points sharing the same Hölder exponent h (Ihlen & Vereijken, 2013; Jaffard et al., 2006).

Hölder Exponents

The function f belongs to class $C^{\alpha}(t_0)$ with $\alpha \geq 0$ if there exists a polynomial P_{t_0} of degree $< \alpha$ and a constant $C > 0$ such that $|f(t_0 + a) - P_{t_0}(t_0 + a)| \leq C|a|^{\alpha}$ as $a \to 0$. The Hölder exponent $h(t_0) = \sup\{\alpha : f \in C^{\alpha}(t_0)\} \geq 0$. Larger $h(t_0)$ indicates smoother local behaviour; values close to zero indicate irregularity. Wavelet leaders are the only multiscale measure proven to accurately characterise the Hölder exponent (Jaffard et al., 2006).

Discrete Wavelet Transform

The DWT of signal $X = \{x_k, k \in \mathbb{Z}\}$ is: $d_x(j,k) = \int X(t)\, 2^{-j}\, \psi_0(2^{-j}t - k)\, dt$, where $\psi(t)$ is the mother wavelet with compact support, j the dyadic scale, and k the translation. Coefficients at scale $j = 1$ are obtained by high-pass filtering and subsampling by 2, repeated for scales $j = 2, 3, \ldots$

Wavelet Leaders

Wavelet leaders $L_x(j,k) = \sup_{\lambda' \in 3\lambda} |d_x(j',k')|$ are the local suprema of wavelet coefficients over three consecutive dyadic intervals $\lambda_{j,k}$ at scale j and all finer scales $j' < j$.

Multifractal Spectrum

The structure function and scaling exponents $\zeta(q)$ are: $S(q,j) = (1/n_j) \Sigma_k |L_x(j,k)|^q$; $\zeta(q) = \lim_{j \to 0} \inf [\log S(j,q) / \log 2^{-j}]$. The scaling exponent $\zeta(q)$ is estimated by linear regression of $\log S(q,j)$ on $\log 2^j$. The multifractal spectrum $D(h)$ is obtained by Legendre transform: $D(h) = \inf_{q \neq 0}(1 + qh - \zeta(q))$. Positive q-orders emphasise large fluctuations (left tail); negative q-orders highlight small fluctuations (right tail) (Ihlen & Vereijken, 2013).

Ihlen, E., & Vereijken, B. (2013). Multifractal formalisms of human behavior. *Human Movement Science*, *32*(4), 633-651. https://doi.org/https://doi.org/10.1016/j.humov.2013.01.008

Jaffard, S., Lashermes, B., & Abry, P. (2006). Wavelet leaders in multifractal analysis. In *Wavelet analysis and applications* (pp. 201-246). Springer.
