# Supplementary material for: Running Constraints Shape Multifractal Movement Complexity Beyond Physiological and Mechanical Load: A Pilot Study
Source: Sensors (Basel). 2026 Sep 17;26(18):5881. doi: 10.3390/s26185881 (PMC13611035; doi:10.3390/s26185881)
Supplement: Supplementary file 1 [file sensors-26-05881-s001.zip › Table S1. Bonferroni-Corrected Pairwise Comparisons for Significant Differences in Heart Rate Variables.pdf]

Table S1. Bonferroni-Corrected Pairwise Comparisons for Significant Differences in Heart Rate Variables.

Exercise Comparison	Mean Difference [95% CI]	Std. Error	*p*	*Cohen's d*
HR $_{SampEn}$ (a.u.)				
CBS x HKR	0.01 [0.00, 0.01]	0.00	0.001	1.163
CBS x SWR	0.00 [0.0, 0.01]	0.00	0.036	0.744
CBS x TER	0.01 [0.00, 0.01]	0.00	0.000	0.827
CBS x TR0%	0.01 [0.00, 0.01]	0.00	0.003	0.934
CBS x TR6%	0.01 [0.00, 0.01]	0.00	0.000	0.993
HR $_{mean}$ (beats·min^{-1})				
CBS x HKR	-21.02 [-31.54, -10,49]	3.45	0.000	-1.431
CBS x TER	-13.59 [-24.36, -2.83]	3.53	0.004	-0.917
CBS x TR6%	-15.86 [-26.17, -5.56]	3.38	0.000	-0.964
HKR x SWR	12.11 [1.59, 22.64]	3.45	0.012	0.825
HKR x TR0%	11.76 [0.77, 22.74]	3.61	0.027	1.168
HR $_{cv}$ (%)				
CBS x HKR	-3.52 [-6.42, -0.62]	0.95	0.007	-0.650
CBS x TER	-3.45 [-6.29, -0.60]	0.93	0.007	-0.916
CBS x TR6%	-3.04 [-6.082, 0.0]	0.99	0.050	-1.074
HKR x TR0%	3.08 [0.05, 6.1]	0.99	0.043	0.506
TER x TR0%	3.01 [0.04, 5.97]	0.97	0.045	1.128

Abbreviations: Coefficient of variation, CV; Core-board skipping, CBS; Heart rate, HR; High-knee running, HKR; Player load, PL; Sample Entropy, SampEn; Shoulder-width running, SWR; Tethered running, TER; Treadmill running at 0%, TR0%; Treadmill running at 6%, TR6%.
