# Supplementary material for: Running Constraints Shape Multifractal Movement Complexity Beyond Physiological and Mechanical Load: A Pilot Study
Source: Sensors (Basel). 2026 Sep 17;26(18):5881. doi: 10.3390/s26185881 (PMC13611035; doi:10.3390/s26185881)
Supplement: Supplementary file 1 [file sensors-26-05881-s001.zip › Table S2. Bonferroni-Corrected Pairwise Comparisons for Significant Differences in Player Load Variables.pdf]

Table S2. Bonferroni-Corrected Pairwise Comparisons for Significant Differences in Player Load Variables.

Exercise Comparison	Mean Difference [95% CI]	Std. Error	*p*	*Cohen's d*
PL_{SampEn} (a.u.)				
CBS x HKR	0.05 [0.02, 0.09]	0.01	0.001	1.200
CBS x SWR	0.04 [0.0, 0.07]	0.01	0.041	0.987
CBS x TER	0.05 [0.01, 0.09]	0.01	0.003	1.535
HKR x TR0%	-0.04 [-0.08, -0.01]	0.01	0.009	-0.880
HKR x TR6%	-0.05 [-0.09, -0.02]	0.01	0.001	-0.762
SWR x TR6%	-0.04 [-0.07, -0.00]	0.01	0.032	-0.874
TER x TR0%	-0.04 [-0.08, -0.0]	0.01	0.031	-1.421
TER x TR6%	-0.05 [-0.09, -0.01]	0.01	0.002	-0.842
PL_{mean} (a.u.)				
CBS x HKR	-0.09 [-0.12, -0.07]	0.01	0.000	-3.087
CBS x SWR	-0.04 [-0.07, -0.02]	0.01	0.000	-1.437
CBS x TER	-0.05 [-0.08, -0.02]	0.01	0.000	-2.285
CBS x TR0%	-0.14 [-0.17, -0.12]	0.01	0.000	-5.467
CBS x TR6%	-0.16 [-0.18, -0.13]	0.01	0.000	-6.178
HKR x SWR	0.06 [0.03, 0.08]	0.01	0.000	1.674
HKR x TER	0.05 [0.02, 0.08]	0.01	0.000	1.262
HKR x TR0%	-0.05 [-0.07, -0.02]	0.01	0.000	-1.752
HKR x TR6%	-0.06 [-0.09, -0.03]	0.01	0.000	-2.085
SWR x TR0%	-0.10 [-0.13, -0.07]	0.01	0.000	-2.435
SWR x TR6%	-0.12 [-0.14, -0.09]	0.01	0.000	-3.112
TER x TR0%	-0.09 [-0.12, -0.06]	0.01	0.000	-2.310
TER x TR6%	-0.11 [-0.13, -0.08]	0.01	0.000	-2.566
PL_{cv} (%)				
CBS x HKR	-18.05 [-30.40, -5.69]	4.06	0.001	-1.297
CBS x SWR	-14.79 [-27.15, -2.44]	4.06	0.008	-0.971
HKR x TR0%	22.09 [9.50, 34.68]	4.13	0.000	1.178
HKR x TR6%	25.13 [12.77, 37.48]	4.06	0.000	1.492
SWR x TR0%	18.84 [6.25, 31.43]	4.13	0.000	1.011
SWR x TR6%	21.88 [9.52, 34.23]	4.06	0.000	1.216
TER x TR6%	13.22 [0.59, 25.84]	4.14	0.033	0.812

Abbreviations: Coefficient of variation, CV; Core-board skipping, CBS; High-knee running, HKR; Player load, PL; Sample Entropy, SampEn; Shoulder-width running, SWR; Tethered running, TER; Treadmill running at 0%, TR0%; Treadmill running at 6%, TR6%.
