# Supplementary material for: Running Constraints Shape Multifractal Movement Complexity Beyond Physiological and Mechanical Load: A Pilot Study
Source: Sensors (Basel). 2026 Sep 17;26(18):5881. doi: 10.3390/s26185881 (PMC13611035; doi:10.3390/s26185881)
Supplement: Supplementary file 1 [file sensors-26-05881-s001.zip › Table S3. Bonferroni-Corrected Pairwise Comparisons for Significant Differences in Muscle Oxygenation Variables.pdf]

Table S3. Bonferroni-Corrected Pairwise Comparisons for Significant Differences in Muscle Oxygenation Variables.

Exercise Comparison	Mean Difference [95% CI]	Std. Error	*p*	*Cohen's d*
SmO_2 GM $_{SampEn}$ (a.u.)				
CBS x HKR	0.01 [0.0, 0.02]	0.00	0.002	0.686
CBS x SWR	0.01 [0.0, 0.02]	0.00	0.002	0.769
CBS x TER	0.01 [0.0, 0.02]	0.00	0.007	0.736
CBS x TR0%	0.01 [0.0, 0.02]	0.00	0.001	0.639
CBS x TR6%	0.01 [0.0, 0.02]	0.00	0.000	0.742
SmO_2 GM $_{mean}$ (%)				
CBS x HKR	15.49 [3.28, 27.70]	3.99	0.004	0.651
CBS x SWR	12.65 [0.44, 24.85]	3.99	0.036	0.543
CBS x TER	12.73 [0.81, 24.66]	3.89	0.027	0.583
CBS x TR6%	13.32 [1.64, 24.99]	3.81	0.014	0.637
SmO_2 VL $_{SampEn}$ (a.u.)				
CBS x HKR	0.01 [0.0, 0.008]	0.00	0.000	1.356
HKR x TER	-0.01 [-0.01, -0.00]	0.00	0.003	-1.581
HKR x TR0%	-0.01 [-0.01, -0.00]	0.00	0.002	-1362
HKR x TR6%	-0.01 [-0.01, -0.00]	0.00	0.003	-0.922
SmO_2 VL $_{mean}$ (%)				
CBS x HKR	13.08 [5.23, 20.92]	2.56	0.000	1.796
CBS x TR6%	10.78 [3.16, 18.40]	2.49	0.001	1.061
HKR x SWR	-8.06 [-15.91, -0.22]	2.56	0.039	-1.147
HKR x TER	-8.07 [-15.73, -0.41]	2.50	0.031	-1.243
HKR x TR0%	-9.91 [-18.35, -1.47]	2.76	0.010	-1.508
SmO_2 VL $_{cv}$ (%)				
CBS x HKR	-29.06 [-38.35, -19.77]	3.04	0.000	-2.568
CBS x TR6%	-19.35 [-28.19, -10.49]	2.89	0.000	-1.468
HKR x SWR	20.74 [11.36, 30.12]	3.07	0.000	1.687
HKR x TER	20.69 [11.19, 30.19]	3.11	0.000	1.709
HKR x TR0%	25.39 [15.66, 35.13]	3.19	0.000	2.110
HKR x TR6%	9.71 [0.42, 19.0]	3.04	0.033	1.160
SWR x TR6%	-11.03 [-20.09, -1.96]	2.97	0.007	-0.970
TER x TR6%	-10.99 [-20.28, -1.69]	3.04	0.009	-0.830
TR0% x TR6%	-15.68 [-25.21, -6.16]	3.12	0.000	-1.412

Abbreviations: Core-board skipping, CBS; *gastrocnemius medialis,* GM; High-knee running, HKR; Shoulder-width running, SWR; Tethered running, TER; Treadmill running at 0%, TR0%; Treadmill running at 6%, TR6%; *vastus lateralis*, VL.
