# Supplementary material for: Running Constraints Shape Multifractal Movement Complexity Beyond Physiological and Mechanical Load: A Pilot Study
Source: Sensors (Basel). 2026 Sep 17;26(18):5881. doi: 10.3390/s26185881 (PMC13611035; doi:10.3390/s26185881)
Supplement: Supplementary file 1 [file sensors-26-05881-s001.zip › Table S4.Detailed description of parameters extracted from the multifractal spectrum.pdf]

Table S4. Detailed description of parameters extracted from the multifractal spectrum (Munro et al., 2018; Zhang et al., 2019)

Multifractal Spectrum Parameters	
$h(0)$	The Hölder exponent corresponding to the point of maximum fractal dimension represents the most frequently occurring singularity. It reflects the central tendency of local fluctuations in the signal.
h_{min}<br>h_{max}	The minimum and maximum values of the Hölder exponent h, respectively. These correspond to the most irregular (left tail) and smoothest (right tail) fluctuations in the signal.
$\Delta h = h_{max} - h_{min}$	The width of the singularity spectrum is used as a measure of the degree of multifractality. A narrow spectrum indicates more uniform, mono-scaling fluctuations (systematic behavior over similar amplitudes). In contrast, a broad spectrum suggests more intermittent and diverse dynamics across a wide range of scales, characteristic of complex multifractal systems.
$Dh(h_{min})$<br>$Dh(h_{max})$	Fractal dimensions associated with the subsets of the signal exhibiting the most irregular and smoothest singularities, respectively.
$\Delta D_h = D_h(h_{max}) - D_h(h_{min})$	Difference between the fractal dimensions of the subsets corresponding to the maximum and minimum probability of occurrence, quantifies the relative probability of different fluctuation magnitudes in the signal. Therefore, a positive ($\Delta D_h > 0$) indicates that higher fluctuation rates occur with greater probability than lower ones, whereas a negative value ($\Delta D_h < 0$) suggests the opposite.
$L = h(0) - h_{min}$<br>$R = h_{max} - h(0)$	The asymmetry degree of the multifractal spectrum. Represent the left (L) and right (R) tail widths of the spectrum. These values indicate the spread of singularities toward the irregular (left) and smooth (right) ends of the spectrum.
$\Delta S = R - L$	The asymmetry degree of the multifractal spectrum. When $\Delta S < 0$, the multifractal spectrum presents a long left tail, indicating that the structure in the time series is not sensitive to local fluctuations with small magnitudes. Conversely, when $\Delta S > 0$ the multifractal spectrum presents a long right tail, indicating that the structure in the signal is not sensitive to the local fluctuations with large magnitude. Additionally, symmetry in the spectrum (i.e., R = L) implies balanced scaling between small and large fluctuations. In case of one of these components exhibits greater complexity, the singularity spectrum becomes asymmetric, causing one tail to extend wider than the other.

Munro, M., Ord, A., & Hobbs, B. (2018). Spatial organization of gold and alteration mineralogy in hydrothermal systems: wavelet analysis of drillcore from Sunrise Dam Gold Mine, Western Australia. *Geological Society, London, Special Publications*, *453*, 165-204. https://doi.org/**https://doi.org/10.1144/SP453.10**

Zhang, X., Liu, H., Zhao, Y., & Zhang, X. (2019). Multifractal detrended fluctuation analysis on air traffic flow time series: A single airport case. *Physica A: Statistical Mechanics and its Applications*, *531*, 121790. https://doi.org/https://doi.org/10.1016/j.physa.2019.121790
