# Supplementary material for: Running Constraints Shape Multifractal Movement Complexity Beyond Physiological and Mechanical Load: A Pilot Study
Source: Sensors (Basel). 2026 Sep 17;26(18):5881. doi: 10.3390/s26185881 (PMC13611035; doi:10.3390/s26185881)
Supplement: Supplementary file 1 [file sensors-26-05881-s001.zip › Table S5.Raw and standardised individual nonlinear PL-derived metrics by running condition and global participant-adjusted condition effects.pdf]

Table S5. Raw and standardised individual nonlinear PL-derived metrics by running condition and global participant-adjusted condition effects.

Metric	CBS	HKR	SWR	TER	TR0%	TR6%	Global effect
**DFA α raw**	0.531± 0.034	0.532± 0.037	0.535± 0.035	0.531± 0.037	0.452± 0.056	0.409± 0.040	F(5,66)=28.17, p<0.001
**DFA α z**	0.53± 0.52	0.55± 0.57	0.60± 0.55	0.54± 0.58	-0.70± 0.88	-1.37± 0.63	same as raw
**Higuchi FD raw**	1.608± 0.039	1.648± 0.025	1.657± 0.046	1.640± 0.048	1.616± 0.037	1.630± 0.040	F(5,66)=4.25, p=0.002
**Higuchi FD z**	-0.59± 0.92	0.37± 0.60	0.57± 1.10	0.18± 1.14	-0.38± 0.87	-0.06± 0.95	same as raw
**PermEn raw**	0.954± 0.008	0.931± 0.012	0.937± 0.013	0.928± 0.017	0.893± 0.028	0.892± 0.023	F(5,66)=28.64, p<0.001
**PermEn z**	1.09± 0.27	0.31± 0.41	0.51± 0.43	0.19± 0.58	-0.99± 0.94	-1.03± 0.79	same as raw

Notes: Values are mean±SD. Standardised values were computed across all available participant-condition PL series. DFA α and Higuchi FD were only modestly correlated (Pearson r = 0.28), supporting their interpretation as complementary scaling descriptors. Abbreviations: Core-board skipping, CBS; High-knee running, HKR; Player load, PL; Shoulder-width running, SWR; Tethered running, TER; Treadmill running at 0%, TR0%; Treadmill running at 6%, TR6%.
