# Supplementary material for: Running Constraints Shape Multifractal Movement Complexity Beyond Physiological and Mechanical Load: A Pilot Study
Source: Sensors (Basel). 2026 Sep 17;26(18):5881. doi: 10.3390/s26185881 (PMC13611035; doi:10.3390/s26185881)
Supplement: Supplementary file 1 [file sensors-26-05881-s001.zip › Table S6.Reduced load-complexity regime dimensions model-estimated condition effects.pdf]

Table S6. Reduced load-complexity regime dimensions: model-estimated condition effects [95% CI].

Dimension	CBS	HKR	SWR	TER	TR0%	TR6%	Model test
Load magnitude	-0.94 [-1.14, -0.74]	0.44 [0.23, 0.65]	-0.39 [-0.60, -0.18]	-0.19 [-0.41, 0.02]	0.41 [0.20, 0.61]	0.69 [0.49, 0.89]	$F(5,65)=37.82$, $p<0.001$
Load variability	-0.43 [-0.72, -0.14]	0.61 [0.30, 0.91]	0.42 [0.12, 0.72]	0.14 [-0.18, 0.45]	-0.47 [-0.77, -0.16]	-0.23 [-0.52, 0.06]	$F(5,65)=9.11$, $p<0.001$
Scaling complexity beyond load	-0.67 [-1.23, -0.11]	0.65 [0.30, 1.01]	0.21 [-0.20, 0.61]	0.12 [-0.26, 0.51]	-0.13 [-0.60, 0.35]	-0.15 [-0.68, 0.37]	$F(5,63)=6.21$, $p<0.001$
Ordinal irregularity beyond load	1.29 [0.68, 1.90]	0.22 [-0.17, 0.60]	0.61 [0.18, 1.05]	0.19 [-0.22, 0.61]	-1.06 [-1.58, -0.55]	-1.20 [-1.77, -0.63]	$F(5,63)=6.50$, $p<0.001$

Notes: Values are estimated marginal means [95% CI] from participant-adjusted fixed-effects models. Load dimensions included condition and participant factors. Complexity dimensions additionally adjusted for standardised HR_{mean} and PL_{mean}. The analysis used 85 matched HR-PL participant-condition observations because one TR0% PL series could not be matched to a corresponding HR trace. Abbreviations: Core-board skipping, CBS; Heart rate, HR; High-knee running, HKR; Player load, PL; Shoulder-width running, SWR; Tethered running, TER; Treadmill running at 0%, TR0%; Treadmill running at 6%, TR6%.
